# Targeting lysosomes in human disease: from basic research to clinical applications

**DOI:** 10.1038/s41392-021-00778-y

**Published:** 2021-11-08

**Authors:** Mengdie Cao, Xiangyuan Luo, Kongming Wu, Xingxing He

**Affiliations:** 1 Institute of Liver and Gastrointestinal Diseases, Tongji Hospital, Tongji Medical College, Huazhong University of Science and Technology, 430030 Wuhan, China; 2 Hubei Key Laboratory of Hepato-Pancreato-Biliary Diseases, Tongji Hospital, Tongji Medical College, Huazhong University of Science and Technology, 430030 Wuhan, China; 3 Department of Medical Oncology, The Affiliated Cancer Hospital of Zhengzhou University & Henan Cancer Hospital, 450008 Zhengzhou, China; 4 Department of Oncology, Tongji Hospital of Tongji Medical College, Huazhong University of Science and Technology, 430030 Wuhan, China

**Keywords:** Molecular medicine, Cancer therapy

## Abstract

In recent years, accumulating evidence has elucidated the role of lysosomes in dynamically regulating cellular and organismal homeostasis. Lysosomal changes and dysfunction have been correlated with the development of numerous diseases. In this review, we interpreted the key biological functions of lysosomes in four areas: cellular metabolism, cell proliferation and differentiation, immunity, and cell death. More importantly, we actively sought to determine the characteristic changes and dysfunction of lysosomes in cells affected by these diseases, the causes of these changes and dysfunction, and their significance to the development and treatment of human disease. Furthermore, we outlined currently available targeting strategies: (1) targeting lysosomal acidification; (2) targeting lysosomal cathepsins; (3) targeting lysosomal membrane permeability and integrity; (4) targeting lysosomal calcium signaling; (5) targeting mTOR signaling; and (6) emerging potential targeting strategies. Moreover, we systematically summarized the corresponding drugs and their application in clinical trials. By integrating basic research with clinical findings, we discussed the current opportunities and challenges of targeting lysosomes in human disease.

## Introduction

**Fig. 1:**
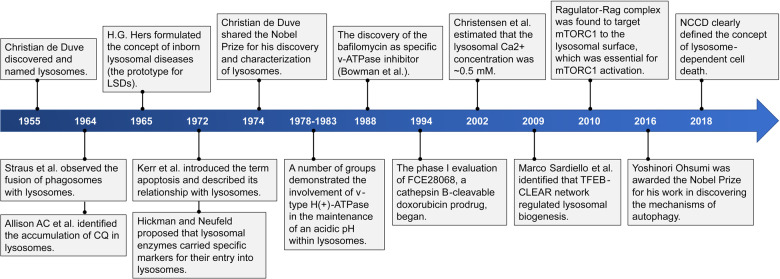
Important events in the development of research into lysosomes as therapeutic targets. Since Christian de Duve discovered and named lysosomes in 1955, scientists have made significant contributions to reveal the structural characteristics and drugs of lysosomes and to connect lysosomes with important pathways such as autophagy, endocytosis, mTOR, and cell death, laying a foundation for the later use of lysosomes as therapeutic targets. Christian de Duve and Yoshinori Ohsumi won the Nobel Prize in 1974 and 2016, respectively, for their contributions to the discovery of lysosomes and the elucidation of autophagy mechanisms. CQ, chloroquine; LSDs, lysosomal storage disorders; CLEAR, coordinated lysosomal expression and regulation; NCCD, Nomenclature Committee on Cell Death

Since Christian de Duve discovered and named lysosomes in 1955, great progress has been made in understanding the structure and function of lysosomes and how they can be harnessed to improve clinical outcomes (Fig. 1).^1–5^ The richness in hydrolytic enzymes is an obvious feature that distinguishes lysosomes from other organelles.^2,6,7^ More than 60 acid hydrolases, which break down cell components and complex macromolecules into their constituent building blocks, have been identified in lysosomes.^2,6,7^ Therefore, lysosomes have long been regarded as “suicide bags” and the “garbage-disposal system” for cells.^2,6^ Nevertheless, in recent years, researchers have gained a better understanding of lysosomes by combining genomics, transcriptomics, proteomics, bioinformatics, and other methods and found that their functions are far more than digestion.^7^ Lysosomes are now regarded as regulators of cell and organismal homeostasis that mediate signal transduction, metabolic adaptation, cell proliferation, cell differentiation, cell secretion, and the quality control of proteins and organelles.^2,6,8^

Lysosomal changes and dysfunction are have profound implications for the development of numerous human diseases.^9,10^ The prevalence of neurodegenerative and cardiovascular diseases in the elderly was thought to be closely related to the decline in lysosomal function with age.^9,11^ In contrast, cancer cells upregulate their metabolism by modulating lysosomal quantity, composition, and activity to meet their needs for cell growth and proliferation.^2,8^ Besides, the translocation and abnormal secretion of lysosomes are conducive to the invasion and metastasis of cancer cells, and upregulated autophagy is considered a vital means by which cancer cells develop resistance to chemotherapy and radiotherapy.^5,10,12,13^ Growing attention has been paid to the role of lysosomes in immunity.^14^ The abnormal degradation of major histocompatibility complex (MHC) molecules and immune checkpoints by lysosomes in cancer cells, as well as the defects in selective autophagy of tumor-infiltrating T lymphocytes, together contribute to tumor immune escape.^15–17^ In the cells of patients with autoimmune diseases, changes in lysosomal biogenesis, acidification and cathepsin activity have also been confirmed, and such changes are thought to be closely related to disease activity and progression.^18–23^

These lysosomal changes and dysfunction play a crucial role in the development of diseases, but they may also provide a therapeutic window for treatment.^9,10,14^ For example, increases in lysosomal size and capacity facilitate cell metabolism but also reduce the stability of lysosomal membranes, making cells more vulnerable to death.^2,24^ In addition to correcting these changes and abnormalities, we may also be able to use them to combat pathological cells.^5,25,26^ In this review, we summarized and discussed recent studies that clarified and supported the idea of targeting lysosomes in human disease and further explored the feasibility, opportunities, and challenges of such efforts by combining basic research with clinical research progress.

## Lysosomal structure, biogenesis, and function

**Fig. 2:**
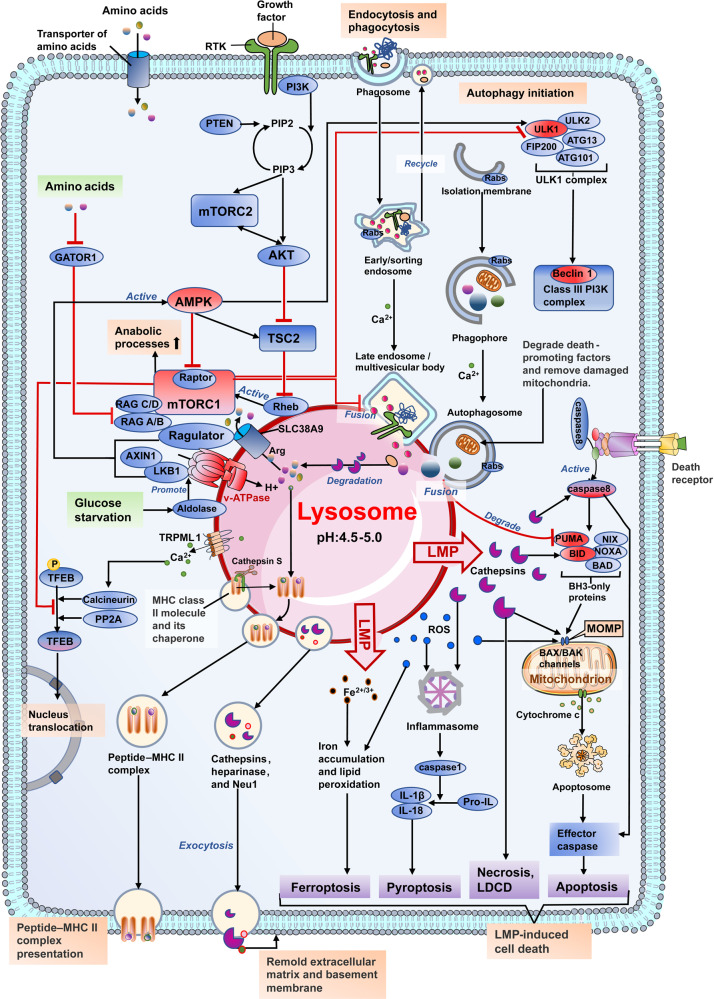
Lysosomal structure and function. The lysosome is an acidic membrane-enclosed vesicular organelle containing a variety of hydrolases, and its activity and function are maintained by the channels or pump structures on its surface, such as v-ATPase, iron channels, and nutrient transporters. The lysosome acts not only as the endpoint of multiple trafficking routes, including autophagy, endocytosis, and phagocytosis, but also, as the platform for the recruitment and activation of mTOR, which regulates cell metabolism, growth, and differentiation. Ca^2+^ released from lysosomal calcium channels such as TRPML also regulates endocytic membrane trafficking, the nuclear transduction of TFEB, and the fusion of lysosomes with other cellular structures, such as autophagosomes and endosomes. However, the leakage of lysosomal contents such as cathepsins, ROS, Fe^2+/3+^, and Ca^2+^ contributes to multiple forms of cell death. Exogenous antigens are processed into peptides by lysosomal proteases, and the lysosome also acts as a bilateral switch that mediate both pro-inflammatory and anti-inflammatory processes. The central location of the lysosome in the communication and convergence of multiple pathways determines its pivotal and irreplaceable role in cell metabolism, proliferation, differentiation, immunity, and death. Black arrows indicate positive regulation or metabolite flux, while red arrows indicate negative regulation. mTORC1, mammalian target of rapamycin complex 1; mTORC2, mammalian target of rapamycin complex 2; Arg, arginine; TSC2, tuberous sclerosis complex; AMPK, AMP-activated protein kinase; TFEB, transcription factor EB; RTK, receptor tyrosine kinases; PP2A, protein phosphatase 2 A; TRPML1, transient receptor potential mucolipin 1; v-ATPase, vacuolar H + -adenosine triphosphatase; HSC70, heat shock cognate protein 70; LAMP2A, lysosome-associated membrane protein 2; LMP, lysosomal membrane permeability; MOMP, mitochondrial outer membrane permeabilization; CMA, chaperone-mediated autophagy; TGN, trans-Golgi network; CLEAR, coordinated lysosomal expression and regulation; ROS, reactive oxygen species; RAG, RAS-related GTP-binding protein; Unc-51-like kinase 1 (ULK1); PUMA, p53 upregulated modulator of apoptosis; BID, BH3 interacting domain death agonist; BAX, BCL2-associated X, apoptosis regulator; LDCD, lysosome-dependent cell death

The lysosome is a membrane-enclosed vesicular organelle that contains two classes of proteins essential for the maintenance of structure and function: soluble lysosomal hydrolases that performs digestive functions and lysosomal membrane proteins with more complex functions, such as acting as proton pump and promoting intercellular interaction (Fig. 2).^2,6,8^ More than 60 acid hydrolases, including proteases, lipases, nucleases, have been found within the lysosome, and they require an acidic (pH ~4.5) environment maintained by the cooperation of an ATP-driven proton pump called the vacuolar H + -adenosine triphosphatase (v-ATPase) with other ion channels.^7,27^ A variety of lysosomal membrane proteins of mammalian cell have been identified, such as lysosome-associated membrane protein 1(LAMP1), LAMP2, lysosome integral membrane protein 2 (LIMP2; also known as SCARB2), v-ATPase, and acid sphingomyelinase (ASM).^6,14,28^ Of these proteins, LAMP1 and LAMP2 are the most abundant (accounting for 50% of all) lysosomal membrane proteins and are essential for metabolism, biogenesis, signal transduction, and cell homeostasis.^2,6,29^

**Fig. 3:**
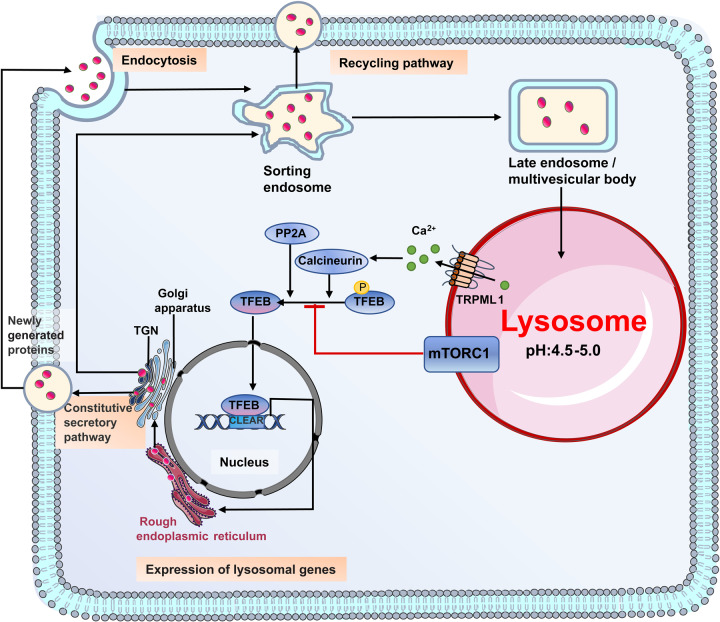
The biogenesis of lysosomes. Lysosomal biogenesis is a combination of cellular biosynthesis and endocytosis pathways. TRPML1 channel, calcineurin, PP2A, and mTORC1 jointly regulate the biosynthesis of lysosomal proteins by modulating the activation and nuclear translocation of TFEB. PP2A, protein phosphatase 2A; mTORC1, mammalian target of rapamycin complex 1; TGN, trans-Golgi network; TFEB, transcription factor EB; CLEAR, coordinated lysosomal expression and regulation

Lysosomal biogenesis is a combination of cellular biosynthesis and endocytosis pathways (Fig. 3).^6,28^ The expression of lysosomal genes is triggered by the binding of transcription factors (TFs) of microphthalmia/transcription factor E (MiT/TFE) family to the coordinated lysosomal expression and regulation (CLEAR) elements.^2,30,31^ Among these TFs, transcription factor EB (TFEB) is the first and most thoroughly studied TF known to directly bind to the CLEAR element.^2,30,31^ Transient receptor potential mucolipin channel (TRPML1), calcineurin, protein phosphatase 2A (PP2A), and mammalian target of rapamycin complex 1 (mTORC1) jointly regulate the activation and nuclear translocation of TFEB by modulating its phosphorylation status.^2,8,32–34^ After being synthesized in endoplasmic reticulum (ER), lysosomal proteins are transported to trans-Golgi network (TGN) and then be secreted to plasma membrane for subsequent endocytosis, or transmitted directly to the endo-lysosomal system.^28^ Sorting events in endo-lysosomal system eventually cause these compartments to be rich in lysosomal membrane proteins and lysosomal hydrolases, which constitute the major components of lysosomes.^8,28^

### Lysosomes in cellular metabolism

Lysosomes are responsible for breaking down and recycling intracellular materials (through autophagy) and extracellular materials (through endocytosis and phagocytosis), which are then used to generate new cellular components and nutrients to meet the needs of cell metabolism and growth (Fig. 2).^2,5,35,36^

Lysosomes serve as the platforms for proper recruitment, assembly, and activation of mammalian target of rapamycin (mTOR) complex 1 (mTORC1), the mediator that coordinates the balance between anabolism and catabolism (Fig. 2).^37,38^ When nutrients are abundant, the stimulation of amino acids such as arginine and glutamine induce the activation of RAS-related GTP-binding proteins (RAGs), which interact with Ragulator and then trigger the recruitment of mTORC1 to lysosomal surface.^4,37,39^ Through the PI3K-AKT pathway, growth factors such as insulin activate Rheb, which binds to mTORC1 on the lysosomal surface and results in its activation.^37,40,41^ In return, mTORC1 inhibits lysosomal biogenesis though phosphorylating TFEB at Ser211 and inhibiting its nuclear transudtion^42–44^ and inhibits autophagy initiation by phosphorylating Unc-51-like kinase 1 (ULK1) at Ser757.^45,46^ The activated mTORC1 signaling also inhibits the lysosomal catabolism of extracellular proteins taken up through the macropinocytosis pathway, a nonselective form of endocytosis.^46,47^

When cells are deficient in nutrients, the inactivation of mTOR signaling and the formulation of the AMP-activated protein kinase (AMPK) complex contribute the upregulation of catabolism pathways.^45^ The deficiency of glucose sensed by aldolase promotes the interaction of axis inhibition protein 1 (AXIN)-live kinase B1 (LKB1) complex with v-ATPase and Ragulator, which then activates AMPK signaling (Fig. 2).^48–50^ Concurrently, AXIN cause the dissociation and inactivation of mTORC1 by interfering with the interaction between RAGs and the Ragulator, and the inactivation of mTORC1 restores lysosome biogenesis and autophagy.^50^ The activated AMPK not only promotes autophagy by phosphorylating the Ser317 and Ser777 of ULK1 but also inhibits mTOR pathway through phosphorylating the Raptor of mTORC1 and activating tuberous sclerosis complex (TSC2), which inhibits Rheb.^45,51,52^ As a scavenging pathway of extracellular proteins, the macropinocytosis pathway was also identified to be upregulated when inhibiting mTORC1.^47,53^ The amino acids obtained through catabolism pathways are then transported out of lysosomes by the lysosomal transmembrane protein called SLC38A9 in an arginine-regulated manner.^54,55^ By interacting with RAGs and Ragulator, SLC38A9 acts as an amino sensor essential for the activation the mTORC1 pathway.^54,56^ The increase of nutrients can lead to the reactivation of mTORC1, thereby inhibiting the catabolic pathway and promoting substance synthesis and cell growth and proliferation.^47,54,56^ When nutrients are scarce, the mTOR signaling is suppressed again.^4,37,39^ Therefore, mTOR signaling coordinates the activity of cell anabolism and catabolism and keeps them in a dynamic balance to meet the needs of cell growth and proliferation.^46^

Taken together, these findings indicate that lysosomes not only play a great role in cellular catabolism, which supplies nutrients for cell growth but also function as a platform for nutrient sensing and metabolic signal transduction (Fig. 2).

### Lysosomes in cell proliferation and differentiation

In addition to mediating the adaptation of cell metabolism to meet the needs for cell growth and proliferation, lysosomes also mediate the turnover of cell surface receptors and other elements crucial to proliferation and differentiation signaling.^2,27,57^ Take epidermal growth factor receptor (EGFR), one of the most well-studied receptor tyrosine kinases (RTKs), as an example.^27^ After binding with ligands and activating downstream pathways, the clathrin adaptor protein complex AP2, growth factor receptor-bound protein 2 (Grb2), epsin, and eps15 together contribute to the endocytosis of EGFR, which then is and sorted for recycling or degradation.^58^ Suppressor of T-cell receptor (TCR) signaling (Sts)-1 and Sts-2 have been found to inhibit the endocytosis of activated EGFR through interacting with ubiquitin ligase Cbl.^59^ Besides, autophagy was reported to facilitate the recycling of EGFR by regulating early endosome homeostasis.^60^ Cells lacking *ATG7* or *ATG16L1*, pivotal autophagy genes, were found to have an accumulation of early endosomal antigen-1 (EEA1)-positive endosomes resulting in the stalled trafficking of EGFR.^60^ Recently, Weber et al.^61^ used unbiased genetic screens and found that lysosomal acidity influenced cell proliferation by maintaining iron homeostasis. While mTORC1 facilitates cell growth and proliferation through promoting cell anabolism, mTORC2 mediates proliferation more directly by phosphorylating and activating the members the AGC (PKA/PKG/PKC) protein kinases, such as AKT, PKCα, PKCδ, PKCγ, and PKCε.^38,62^ It is worth noting that both mTORC2 and AKT signaling were found to be influenced by lysosome positioning.^63^

It has been proved that mTOR signaling (both mTORC1 and mTORC2) plays a pivotal role in the regulation of differentiation and function of numerous immune cells, such as T lymphocytes, macrophages, dendritic cells (DCs), and adipocytes.^64–70^ Mtor^−/−^T cells have been shown to be unable to differentiate into Th1, Th2 and Th17 cells, despite having normal activation markers and levels of IL-2 production under T-cell receptor (TCR) stimulation.^71^ Although mTOR signaling facilitates cell growth and proliferation, sustained activation of mTOR signaling has been found to result to the terminal differentiation and reduced proliferative capacity of T cells.^72–75^ Besides, auophagy plays different roles in cell differentiation, which may be correlated with the difference of stimulation and the stage of cell differentiation.^69,76,77^ While the differentiation of peripheral blood monocytes into macrophages induced by colony stimulating factor 1(CSF-1) was identified highly dependent on autophagy, Zhang et al.^69^ found that the inhibition of autophagy promoted the macrophagic differentiation of myeloid hematopoietic progenitor cells.^76,77^

Taken together, these findings suggest that the lysosome is a crucial regulatory hub for multiple pathways involved in cell proliferation and differentiation (Fig. 2).

### Lysosomes in immunity

It is well-established that exogenous antigens are primarily processed into peptides by lysosomal proteases and loaded onto MHC-II molecules for recognition.^14,78^ In recent years, the high expression of MHC-II molecules has also been found to be correlated with a favorable prognosis of cancer patients.^79^ While autophagy facilitates the loading of MHC class II molecules by delivering cytoplasmic components to lysosomes, it also interferes with antigen presentation by MHC-I molecules through competing ubiquitinated proteins with proteasomes and degrading proteasomes.^80–83^ Expansion of the volume and protein levels of lysosomes and endosomes in phagocytes was found to promote antigen presentation in the immune response, and lysosomal recruitment and secretion was identified to facilitate the antigen extraction and the full activation of B lymphocytes.^84–86^ In addition to their role in antigen processing and presentation, lysosomes are responsible for the degradation and membrane presentation of immune checkpoints such as programmed cell death-ligand 1 (PD-L1),^16,87^ cytotoxic T-lymphocyte-associated protein 4 (CTLA-4),^87^ and CD70.^10,88^ Abnormalities in the degradation and presentation of immune checkpoints are closely associated with the progression and treatment failure of many diseases, especially cancer.^16,87,89^ After antigen stimulation, mTOR signaling is activated and then programs the differentiation of immune cells into functionally distinct lineages, such as Th1, Th2, Th17, Treg, cytotoxic CD8 + T cells, and memory CD8 + T cells.^64,70,90^

Lysosomes act as bilateral switches that regulate inflammatory process, an important part of the immune response that has both protective and disease-driving roles.^14,30,91–93^ On the one hand, lysosomes mediate the release of pro-inflammatory cytokines from immune cells, and the release of lysosomal hydrolase are of great benefit to the initiation and development of inflammation.^30,92^ Lysosomes have also been found to promote inflammation by degrading glucocorticoid receptors, which bind with glucocorticoid to modulate the expression of and inflammatory and pro-inflammatory factors.^94,95^ On the other hand, lysosomes mediate the release of anti-inflammatory cytokines and are responsible for breaking down inflammatory cytokines and elements such as PYCARD/ASC, a critical component of inflammasome.^2,30,96^

Taken together, these findings show that lysosomes are closely involved in the immune response and its strength modulation, and serve as the two-way switches that regulate inflammation (Fig. 2).

### Lysosomes in cell death

Lysosomes mediate cell death at several levels. Under some unfavorable conditions, autophagy is activated to avoid cell death by degrading death-promoting factors such as (p53 upregulated modulator of apoptosis) PUMA and receptor-interacting protein kinases-1 (RIPK1) and promoting autophagy-dependent mitochondrial homeostasis.^5,97,98^ When exposed to extreme stress, lysosomal membrane permeability (LMP) occurs, and the leakage of cathepsins, reactive oxygen species (ROS), and Fe^2+/3+^ triggers multiple forms of cell death, such as apoptosis, necrosis, pyroptosis, ferroptosis, and lysosome-dependent cell death (LDCD) (Fig. 2).^2,24,26,99^ For instance, while cathepsins leaked from lysosomes can promote apoptosis through activating BID proteins or BAX channels, robust lysosomal cathepsin activities lead to cell necrosis by rapidly degrading essential cell components.^100,101^ Different from chaotic and drastic degradation of cell components that occurs in necrosis, LDCD was defined by the Nomenclature Committee on Cell Death (NCCD) as the regulated cell death demarcated by primary LMP and precipitated by cathepsins with or without the involvement of caspases and mitochondrial outer membrane permeabilization (MOMP).^102^

Although autophagy acts as a cytoprotective process most of the time, the pathway and its key components also participate lethal signaling.^26,98,101,103^ For example, it has been acknowledged that selective autophagy can promote ferroptosis through degrading ferritin and intracellular lipid droplets, causing iron accumulation and lipid peroxidation.^103–105^ Although much remains unclear, it has been confirmed that lysosomes play a crucial role in resisting and triggering cell death and the terminal clearance stage of cell death (Fig. 2).

## Characteristic changes and dysfunction of lysosomes in human disease

Given the important roles of lysosomes in cell metabolism, cell proliferation and differentiation, immunity, and cell death, any lysosomal change or dysfunction may disrupt original cell and organismal homeostasis, causing or deteriorating human disease. As early as the 1960s, H.G. Hers discovered the relationship between the deficiency in lysosomal α‑glucosidase and Pompe disease and first proposed the concept of inborn lysosomal disease, the prototype of lysosomal storage disorders (LSDs) (Fig. 1).^106,107^ LSD are a group of rare metabolic disorders caused by inherited defects in genes that encode proteins involved in lysosomal homeostasis, such as lysosomal hydrolases or membrane proteins.^108,109^ In addition to LSDs, the initiation and development of numerous diseases, such as cancer, autoimmune disorders, neurodegenerative diseases, and cardiovascular diseases, have also been identified to have a close correlation with lysosomal changes and dysfunction.^110–129^ In this section, we started with several diseases and studied their lysosomal changes and dysfunction to lay the foundation for the selection of targeted strategies.

### Cancer

Cancer cells always upregulate their metabolism by changing the quantity, localization, and activity of lysosomes to meet their needs for cell growth and proliferation.^2,8^ These changes have been correlated with the overexpression of some lysosomal proteins and lysosome-related proteins, such as lysosome catalase, lysosomal glycosidase, and kinesins.^10,130–132^ The abnormal activation of classical oncogenes, such as *Kras* and *MYC*, was found to increase the expression of lysosome catalase and glycosidase.^10^ Furthermore, several types of cancer, such as pancreatic adenocarcinoma,^110,111^ renal-cell carcinoma,^110^ melanoma,^110,112^ and breast cancer,^113^ have been found to have an overexpression of *MIT/TFE* genes, the TFs that facilitate the expression of lysosomal proteins.^2^

These lysosomal changes have profound effects on the proliferation, migration, and invasion of cancer cells, as well as their resistance to radiotherapy and chemotherapy.^2,8,27^ Upregulated nutrients-scavenging pathways such as autophagy and endocytosis allow cancer cells to compete for available nutrients and survive in unfavorable conditions, such as tumors with poor vascularization or undergoing radiotherapy or chemotherapy.^8^ Nutrients brought by upregulated nutrient-scavenging pathways activate mTOR signaling and promote cell synthesis of amino acids, glucose, nucleotides, fatty acids, and lipids, which are essential for cell proliferation.^8,37,133^ Aberrant hyperactivation of both catabolic and anabolic pathways facilitates the metabolism and proliferation of cancer cells.^2^ MTORC1 signaling and TFEB modulation constitute a feedback loop that coordinates the balance between lysosomal catabolism and anabolism to adapt to different metabolic conditions.^2^ In addition, lysosomes have also been found to contribute to the chemoresistance of cancer cells by sequestering drugs to prevent their action outside lysosomes.^12^ Besides, the upregulated autophagy pathway favors the invasion and metastasis of cancer cells through degrading epithelial-derived molecules such as E-cadherin.^5,10^ Furthermore, the redistribution of lysosomes to the periphery of cancer cells and their exocytosis of cathepsins, heparinase, and Neu1 also benefit cancer invasion, metastasis, and angiogenesis by affecting cell morphology and degrading their extracellular matrix and basement membrane.^2,27^

Lysosomal changes and dysfunction also play an important role in the escape of cancer cells from the host immune system. Lysosomal degradation is not only responsible for antigen processing but also controls the presentation of MHC-I at cell membrane.^134–136^ It has been reported that lysosomal degradation of MHC-I through autophagy-dependent pathways accounts for the decreased expression of MHC-I on the cell surface of pancreatic ductal adenocarcinoma (PDAC).^135^ The co-location of MHC-I with autophagosomes and lysosomes was observed in PDAC cells, and autophagy inhibition was identified to restore MHC-I levels and promote T-cell responses in mouse models.^135^ Besides, the deficiency or blockade of costimulatory molecules of tumor cells is one of the important mechanisms of tumor immune escape, and lysosomes are responsible for not only the degradation but also the membrane transportation and presentation of immune checkpoints such as CTLA-4, PD-L1, and CD47.^10,27,89^ The colocalization of CKLF-like MARVEL transmembrane domain-containing 6 (CMTM6) with PD-L1 in cell membrane and recycling endosomes was found to inhibit the lysosomal degradation of PD-L1, which interacts with PD-1 on T cells to evade T-cell-mediated immunosurveillance.^16^ Recently, the decreased activity of mitophagy, a type of selective autophagy, has been reported to lead to the accumulation of depolarized mitochondria in tumor-infiltrating T lymphocytes (TILs).^17^ The persistent metabolic insufficiency caused by defective mitophagy was thought to cause TLR exhaustion.^17^ These factors together contribute to the low immune response in tumors.

**Fig. 4:**
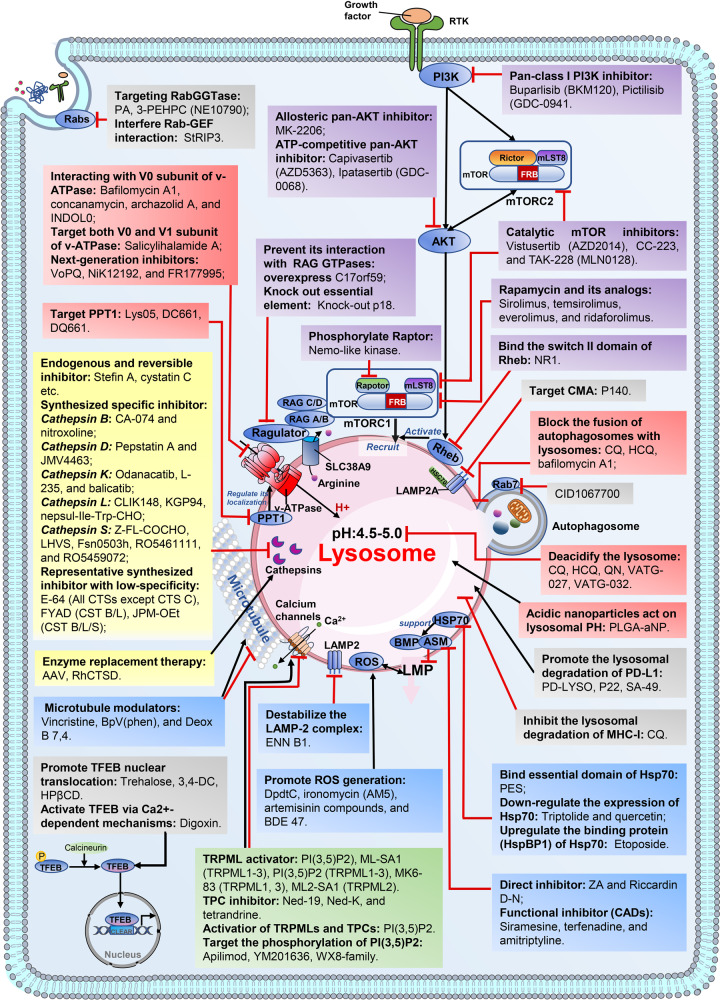
Available strategies for targeting lysosomes in human disease and their corresponding drugs. Different colors indicate different targeting strategies: red, targeting lysosomal acidification; yellow, targeting lysosomal cathepsins; blue, targeting lysosomal membrane permeability and integrity; green, targeting lysosomal calcium signaling; purple, targeting mTOR signaling; gray, emerging targeting strategies with great potential. The action mechanisms of these drugs are highlighted in bold. mTORC1, mammalian target of rapamycin complex 1; mTORC2, mammalian target of rapamycin complex 2; TRPML1, transient receptor potential mucolipin 1; LMP, lysosomal membrane permeability; ROS, reactive oxygen species; CMA, chaperone-mediated autophagy; CLEAR, coordinated lysosomal expression and regulation; TFEB, transcription factor EB; CQ, chloroquine; HCQ, hydroxychloroquine; QN, quinacrine; PLGA-aNP, poly(DL-lactide-co-glycolide) acidic nanoparticles; AAV, adeno-associated virus; rhCTSD, recombinant human pro-cathepsin D; rhPPCA, recombinant human protective protein/cathepsin A; ASM, acid sphingomyelinase; ZA, zoledronic acid; rhCTSD, recombinant human pro-Cathepsin D; DpdtC, Di-2-pyridylketone dithiocarbamate; Hsp70, heat shock protein 70; HspBP1, Hsp70 binding protein 1; 3,4-DC, 3,4-dimethoxychalcone; PI(3,5)P2, phosphatidyl-(3,5)-bisphosphate; MITF, melanogenesis-associated transcription factor; HPβCD, 2-Hydroxypropyl-β-cyclodextrin; PA, psoromic acid; 3-PEHPC, 3-(3-pyridyl)-2-hydroxy-2-phosphonopropanoic acid; RabGGTase, Rab geranylgeranyl transferase

However, the changes mentioned above not only benefit cancer development but also lead to the reduced stability of lysosomal membranes and make lysosomes in cancer cells more susceptible to LMP, which may provide the therapeutic windows we seek.^2,24,26,137^ Considering the great role of these changes and dysfunctions in lysosomes in cancer cells, it is feasible to develop strategies targeting lysosomes to treat cancer (Fig. 4).

### Autoimmune disorders

Given that lysosomes play a crucial role in multiple stages of immunity, we took a close look at what happened to the lysosomes in the cells of patients with autoimmune disorders. Here, we take three kinds of rheumatic autoimmune disorders as examples to explain lysosomal changes and dysfunction in autoimmune disorders and the significance of these changes and dysfunction for the deterioration of these disorders.

One of the most obvious changes in the cells of autoimmune disorders is the upregulated expression and activity of cathepsins and their abnormal secretion (The details are provided in Table 1).^14,114–119,138^ The overexpression of cathepsin S, an enzyme responsible for degrading antigens, has been reported in all three diseases, and its inhibitors have been shown to be effective in mouse models.^14,114–118,138^ In addition, cathepsin S and L were found to have significant correlations with rheumatoid arthritis (RA)-associated autoantibodies, which may account for the chronic inflammatory response and destruction of human tissues.^139^ More directly, cathepsin B present in synovial fluid of the joint of patients with RA has been found to destroy joints by degrading bone collagen.^140^

**Table 1:** Characteristic changes and dysfunction of lysosomes in autoimmune disorders

Disease	Lysosomal biogenesis and acidification	Lysosomal cathepsin	Autophagy
SLE	• The lysosomal pH of **monocytes, B cells, and DCs** from female patients is lower than that of male patients because of higher the expression of CXorf21;^21,154^ • **Macrophages** from lupus-prone MRL/lpr mice were reported to have attenuated lysosomal acidification.^155^ (contrast)	• **Cathepsin S** was found to be upregulated in MRL/lpr mice, and its inhibitor RO5461111 suppressed the plasma levels of numerous IgG (but not IgM) autoantibodies including anti-dsDNA;^115^ • Elevated serum level of **cathepsin K** was found in MRL/lpr mice, and cathepsin K deficiency was reported to ameliorate their SLE-like manifestations;^375^ • Plasma **cathepsin B** level was found causally associated with SLE.^326^	• Autophagy was found activated in the B lymphocytes of the NZB/WF1 murine lupus model and acted as a survival mechanism for **autoreactive B cells**;^141^ • **Autophagy-related genes** such as *ATG5*, *CDKN1B*, *DRAM1*, *CLEC16A*, and *ATG16L2* were reported to be associated with susceptibility to SLE;^20^ • Elevated autophagic vacuoles were found in the **peripheral T cells** of lupus-prone mouse models and lupus patients;^376^ • Upregulated autophagy in **macrophages** contributed to murine lupus by promoting the production of pro-inflammatory cytokines TNF-α and IL-6;^142^ • Increased autophagy protected **podocytes** from injuries that were induced by antibody and interferon-α in lupus nephritis.^377,378^ Mice with defective **LC3-associated phagocytosis pathway** showed increased serum levels of inflammatory cytokines and autoantibodies and evidence of **kidney damage**;^379^
RA	**Lysosomal biogenesis:** upregulated expression of TFEB;^380^ **Lysosomal acidification:** neutrophils in patients showed lower lysosomal pH.^145^	• Upregulated expression of **cathepsin S**;^138^ • Upregulated mRNA expression of **cathepsin K** in synovial fibroblasts;^381^ • **Cathepsin B** present in synovial fluid of the joint of patients lead to joint destruction by degrading collagen;^140^ • **Cathepsin S and L** have been found to have significant correlations with RA-associated autoantibodies;^139^ • The level of **cathepsin G** was fund to raise in the synovial fluid of patients and participate in joint inflammation through its chemoattractant activity.^382^	• Autophagy was upregulated in **synovial tissues** of patients with active RA and correlated with disease severity;^383^ • Autophagy induction was reported to promote survival of **fibroblast-like synovial cells** from RA patients under endoplasmic reticulum stress and methotrexate treatment;^143,144^ • Autophagy was activated in **osteoclasts** from human RA patients in a TNFα-dependent manner and regulated osteoclast differentiation and bone resorption;^149^ • **Neutrophils** in patients showed upregulated expression of autophagy-related LC3 protein;^145^ • **Increased** autophagy was reported in total **CD4** + **T cells** of RA patients, which result in T-cell hyperactivation and apoptosis resistance;^151^ An autophagy **defect** associated with PFKFB3 deficiency was observed in naive CD4 + T cells of RA patients;^152^
SS	Not determined.	• Increased expression of **cathepsin S** in lacrimal gland;^114^ Imbalanced Rab3D vs. Rab27 caused the increased **cathepsin S secretion** from lacrimal acini;^119^ • The expression of **cathepsin B and D** in minor salivary glands were upregulated by prolactin.^384^	• Upregulated autophagy was observed in **T and B cells infiltrating SS minor salivary glands**, and was associated with histological severity;^19,146,147^ • Increased level of autophagy markers (ATG5 and LC3B-II) have been identified in **tears and conjunctival epithelial cells** of patients with primary SS; • Defective macroautophagy and chaperone-mediated autophagy have been observed in the **salivary glands of MRL/lpr mice** that develop a secondary SS.^385^

*SLE* systemic lupus erythematosus, *RA* rheumatoid arthritis, *SS* Sjögren’s syndrome, *DCs* dendritic cells, *TFEB* transcription factor EB

Another change that greatly arouses our attention is the dysregulation of autophagy. Enhanced activation of autophagy within autoreactive cells and inflammatory cells is common in systemic lupus erythematosus (SLE), RA, and Sjögren’s syndrome (SS).^19,141–147^ It has been found that autophagy is not only the survival mechanism of autoreactive B cells in SLE patients, but also the key to plasmablast differentiation and the long-term autoantibody secretion of plasma cells.^141,148^ In patients with RA, upregulated autophagy was found to regulate the bone resorption of osteoclasts and promote the survival of fibroblast-like synovial cells, the main actor in RA pathogenesis.^143,144,149,150^ Upregulated autophagy was also observed in T and B cells that infiltrated minor salivary glands in SS, and was associated with histological severity.^19,146,147^ However, there are different views regarding the activation status of the autophagy pathway in the T cells of RA patients.^151,152^ While Yang et al. identified the autophagy defect associated with PFKFB3 deficiency in CD4 + T cells of RA patients, van Loosdregt et al.^151^ reported that autophagy in CD4 + T cells of RA patients was upregulated and promoted hyperactivation and apoptosis resistance of T cells.^152^ It is important to point out that the former study used naive CD4 + T cells while the latter used total CD4 + T cells. Therefore, the differences in the cell type might contribute to the difference in the results. In addition, Gros et al.^153^ supported the view that autophagy was upregulated in T cells, and believed that the reason for the difference in results might be the imperfect experimental design of the former study.

In theory, upregulated lysosomal biogenesis and reduced lysosomal pH are required for the maintenance of cathepsin activity and autophagy activation. Consistent with our expectation, the monocytes, B cells, and DCs from female SLE patients showed lower lysosomal pH than those of normal people.^21,154^ Nevertheless, while upregulated autophagy in macrophages has been reported to contribute to murine lupus, another study reported that macrophages from lupus-prone MRL/lpr mice exhibited impaired lysosomal maturation and acidification.^142,155^ Currently, there are few reports on the changes on lysosomal biogenesis and pH, and more research results are needed to clarify these issues.

Although much is still unknown about the lysosomal changes that occur in the cells of patients with autoimmune disorders, several lysosomal targeting agents have been shown to be effective in preclinical and clinical trials, such as P140, hydroxychloroquine, and RO5461111 (Fig. 4 and Supplementary Table S1).^139,140,153,156^ Therefore, targeting lysosomes in autoimmune disorders is still of great feasibility and potential.

### Neurodegenerative diseases

The accumulation of modified or misfolded proteins is common in neurodegenerative diseases, such as Alzheimer’s disease (AD), Parkinson’s disease (PD), and Huntington’s disease (HD).^14,157^ These proteins not only deposit in neurons, causing synapse destruction and neuronal death, but also impair lysosomal function, which aggravates their accumulation.^157–161^ For instance, α-synuclein, the main component of Lewy bodies in PD, has been reported to reduce lysosomal degradation capacity by disrupting hydrolase trafficking.^14,162^ In addition, increased oxidative and nitrative modifications of v-ATPase with age, as well as mutations in *PS1,*^120^ *ATP6AP2,*^121^ and *ATP13A2/PARK9*^122^ have been identified to promote neurodegenerative diseases by impairing lysosomal acidification and autophagy (reviewed elsewhere.^160,163^) Genetic or pharmacological activation of TFEB was shown to partially restore the degradation of misfolded proteins and ameliorate disease progression of AD,^164–166^ PD,^167,168^ and HD.^169,170^ In addition, chaperone-mediated autophagy (CMA) has been found to act as a compensatory pathway of macroautophagy that is activated in response to macroautophagy failure.^171–174^

The expression of cathepsins, the important executors of lysosomal degradation, was deregulated in cells of neurodegenerative diseases, and they showed reduced efficiency in degrading misfolded proteins and deregulated expression.^14,175^ For example, cathepsin D, the hydrolase responsible for degrading HTT, was identified to be less effective in degrading mHTT, the aggregation- prone HTT mutant in HD.^14,176^ It is worth noting that cathepsins do not always play a protective role. The accumulation of Aβ peptides and hyperphosphorylated Tau are hallmarks of AD, and cathepsin D is responsible for the generation of Aβ peptides and be correlated with the hyperphosphorylation of Tau.^157,177,178^ In addition, elevated cathepsin B in the serum has been shown to be identified significant correlated with cognitive dysfunction in patents with AD.^175,179^

Taken together, increasing evidence indicates the great role of lysosomal changes and dysfunction in the pathogenesis of neurodegenerative diseases. Therefore, they may serve as targets for the treatment neurodegenerative diseases.

### Cardiovascular diseases

Accumulating studies have provided insights into lysosomal changes and dysfunction in cardiovascular diseases.^123–129^ During acute myocardial ischemia, upregulated autophagy protects cardiomyocytes from death in response to extreme hypoxia and nutritional stress.^123^ However, an increased abundance of autophagosome has been reported to contribute to cardiomyocyte death during ischemia reperfusion.^124^ Reactive oxygen species (ROS) released during the restoration of blood flow are thought to induce LAMP2 decline and BECLIN-1 upregulation, resulting in the impaired autophagosome clearance.^124^ Impaired autophagic flux and inadequate autophagosome clearance have also been confirmed to promote atherosclerosis,^125,126^ maladaptive post-infarction remodeling,^127,128^ and heart failure^129^ (reviewed elsewhere.^160,180^) In addition, the restoration of autophagosome clearance through forcing TFEB expression have been found to attenuate BNIP3-induced cardiomyocyte death.^181^ Upregulation of cathepsin D induced by myocardial infarction was reported to protect against cardiac remodeling and heart failure through promoting autophagic flux.^182^

Lysosome changes and dysfunction have also been characterized in hereditary cardiomyopathy and drug-induced cardiomyopathy. In the hearts of patients with Danon disease, an X-linked lysosomal storage disease characterized by life-threatening hypertrophic cardiomyopathy, cardiomyocytes showed a dramatically increase in autophagic vacuoles.^183^ It is generally believed that this increase in vacuoles is due to the loss-of-function mutations in the *LAMP2* gene, which encodes the protein required for the maturation of autophagosomes.^183^ Drug-induced cardiotoxicity remains a major cause of concern in the application of numerous medicines.^184^ In recent years, a large number of studies have confirmed that the cardiotoxicity caused by doxorubicin, a classical chemotherapeutic drug, is related to its inhibition of cardiac autophagy via the impairment of lysosomal acidification and the suppression of TFEB expression.^185–187^ Li et al.^185^ posited that ROS accumulation resulting from compromised autophagy accounts for doxorubicin-induced cardiomyocyte injury.

In summary, many cardiovascular diseases have been recognized to be closely related to lysosomal alterations and dysfunction, which are mainly manifested as maladaptive autophagy. Exogenous supplementation with lysosomal enzymes or efforts to increase the expression of TFEB may be means to treat these diseases.

Overall, lysosomes in cancer cells, autoimmune disease cells, heart disease cells and neurodegenerative disease cells undergo some changes and disfunction, which are extremely important for the development of these disease. Lysosomal acidification, lysosomal cathepsins, lysosomal biogenesis and autophagy may serve as good targets for the treatment of these diseases.

## Available strategies for targeting lysosomes in human disease

### Target lysosomal acidification

The acidic environment in lysosomes is not only their structural characteristics but also the basis of their activities and functions.^2,7,188^ While low lysosomal pH is required for cancer cells to maintain their high metabolic state and is associated with the overactivation of autoimmune cells in autoimmune disorders, cells in neurodegenerative and cardiovascular disease always exhibit impaired lysosomal acidification and autophagy.^163,185,189^ Therefore, suitable targeting measures can be selected according to different lysosomal acidification states.

At present, agents inhibiting lysosomal acidification can be roughly divided into two categories: v-ATPase inhibitors and antimalarials and their derivatives (Fig. 4). The v-ATPase contains two essential domains, V0 and V1 domian, which function together to pump protons into the late endosome/lysosome.^188,190^ v-ATPase inhibitors including bafilomycin A1, concanamycin, archazolid A, and INDOL0 all exhibit great performance, but they compete with one another because they all work through interacting with V0 subunit c of v-ATPase.^189,191,192^ In addition, bafilomycin A1 was found to block autophagosome-lysosome fusion by targeting endoplasmic reticulum (ER) calcium pump Ca-P60A independent of v-ATPase-mediated lysosomal acidification.^193,194^ Salicylihalamide A acts through inhibiting the V0 domain and causing a dramatic redistribution of the V1 domain, allowing it to combine with the former class inhibitors to achieve better inhibition.^189,195^ The next generation of inhibitors including VoPQ, NiK12192, and FR177995 also exhibit effective v-ATPase inhibition, but their inhibition efficiency has only been confirmed in yeast cells.^189,196^

Another class of inhibitors is antimalarials and their derivatives, the only class of autophagy inhibitors that is currently available for clinical application.^5,197^ As representative cationic amphiphilic drugs (CADs), CQ and its derivatives share a common structure of a side-chain with a cationic amine group, which allows them to accumulate within lysosomes after protonation, resulting in lysosomal deacidification.^5,26,198^ In addition, CQ and HCQ have shown a strong ability of blocking the fusion of autophagosomes with lysosomes, which may be their main mechanism of autophagy inhibition.^5,199^ Nevertheless, their widespread application is restricted by their excessive dose-dependent effects, limited single-agent activity, and ocular toxicity at high doses or with long-term use.^5,26,200,201^ Quinacrine, another drug originally used for antimalarial therapy, show a 60-fold higher potency of lysosomal deacidification than CQ and may be a better candidate for autophagy inhibition.^8^ Their analogs and derivatives exhibit more potent abilities of lysosomal localization and autophagy inhibition than their prototype, and some derivatives, such as DQ661 and DC661, show additional mTORC1 inhibition.^5,133,200,202–204^ More importantly, Ravi K. Amaravadi et al. identified a target shared by monomeric and dimeric CQ derivatives called palmitoyl-protein thioesterase 1 (PPT1) and demonstrated that targeting PPT1 produced dramatic lysosomal deacidification and mTOR inhibition by modulating the lysosomal localization of v-ATPase subunits and disrupting the interaction between subunit V1A of v-ATPase and the Ragulator component called p18.^203,204^ Intriguingly, there are many similarities between retinopathy caused by long-term use of CQ and HCQ and retinopathy caused by *PPT1* deficiency, such as maculopathy with pigmentary alterations and the presence of auto-fluorescent material throughout the retina.^201,205^ Therefore, there is a question of whether the use of these derivatives will cause more serious ocular side effects due to their stronger inhibition of PPT1.

The restoration of lysosomal acidification in cells with impaired autophagy can be achieved by targeting molecules that impede lysosomal acidification or exogenously supplementing acid.^120,206^ As we described above, mutated *PS1* impaired lysosomal acidification through impeding V0a1 subunit of v-ATPase complex, which is one of the main causes of early-onset familial AD.^120^ Therefore, targeting mutated PS1 may partially restore lysosomal acidity and autophagy. Besides, Bourdenx et al.^206^ demonstrated that poly(DL-lactide-co-glycolide) acidic nanoparticles (PLGA-aNPs) were internalized into lysosomes within 24 h after the treatment and restored defective lysosomal acidification and autophagy-lysosomal pathways in three different pathological PD models, including fibroblasts from PD patients with *ATP13A2* mutations, fibroblasts from PD patients with glucocerebrosidase (*GBA)* mutations, and BE-M17 cells with *ATP13A2* knockdown.

In brief, lysosomal acidification plays different roles in different diseases, and suitable targeting measures can be chosen according to the lysosomal acidification state.

### Target lysosomal cathepsins

Lysosomal cathepsins are among the most important components and functional executors of lysosomes.^2,132^ Accumulating findings have acknowledged that lysosomal cathepsins facilitate the proliferation, invasion, angiogenesis, and chemotherapy-resistance of cancer cells, and their expression and activities are frequently upregulated in leukemia and various solid tumors, such as melanoma, breast cancer, gastrointestinal cancer.^160,207,208^ In addition, as mentioned above, deregulated cathepsins also play a great role in the development and progression of autoimmune disorders and neurodegenerative diseases. Therefore, cathepsins have been proposed as good targets for the treatment of cancers, autoimmune disorders, and neurodegenerative diseases.^22,207,209,210^

Three families and 15 classes of cathepsins have been found in humans, and cathepsin B, cathepsin D, cathepsin K, cathepsin L, and cathepsin S are well-studied in the treatment disease.^14,27,132,207,211,212^ A variety of endogenous and reversible inhibitors show therapeutic potential in regulating cathepsins, such as stefin A and cystatin C.^207,209^ Besides, while the inhibitory effect of most synthesized cathepsin inhibitors is broad-spectrum and irreversible (shown in Fig. 4 and Supplementary Table S1), inhibitors such as CA074, odanacatib (MK-0822), KGP94, CLIK-148, and CLIK-195, are designed to have better specificity and efficiency.^207,213^ Cathepsin K is highly effective at degrading collagens of bone matrix, and its inhibitor odanacatib was once regarded as the most promising candidate for the treatment of bone destruction caused by inflammatory diseases and cancers.^214,215^ However, the development of odanacatib for the treatment of osteoporosis was finally terminated by the study’s sponsor because of its serious cardio-cerebrovascular adverse reactions observed in the phase III clinical trial of postmenopausal osteoporosis.^215,216^ The use of cathepsin antibodies or targeting cathepsin secretion also holds great promise as therapeutic agents to target abnormal activities of lysosomal cathepsins.^2,27,207,217,218^ For example, the nonreceptor tyrosine kinases Abl and Arg (Abl/Arg) were reported to promote the secretion of cathepsin B and cathepsin L, which facilitate melanoma invasion and metastasis by cleaving or degrading extracellular matrix proteins.^218^

As we described above, cathepsin D plays a protective role in HT and cardiac remodeling, so forced expression or exogenously supplementation of cathepsin D may be helpful for the alleviation of these diseases.^176,182^ Two studies in neuronal ceroid lipofuscinosis, a group of rare recessive lysosomal storage disorders with impaired lysosome-autophagy pathways, have provided some direction.^219,220^ The injection of adeno-associated virus encoding mouse cathepsin D into both cerebral ventricles and peritoneum have been proved to increase the lifespan of cathepsin D-knockout mice (*Ctsd−/*− mice).^219^ In addition, André R. A. Marques administered 25 mg/kg recombinant human pro-cathepsin D to *Ctsd−/−* mice through the tail vein and found a correction of lysosomal storage accumulation and impaired autophagic flux in their viscera and central nervous system.^220^ The lifespans of these mice were also longer than those of the control group.^220^ These data support the feasibility and efficiency of restoring lysosomal cathepsins in diseases characterized by reduced cathepsin efficiency.

In recent years, a variety of drugs have been synthesized, but few of them have been used in clinical studies (summarized in the clinical trial section). The complexity of the cathepsin web and our inadequate understanding of the integration and functionality of cathepsins within the web make it difficult to target cathepsins for clinical application.^132,207,213^ In addition, enzyme replacement therapy is not yet mature, and it is difficult to achieve accurate and efficient delivery of cathepsins to specific organs.^108^

### Target lysosomal membrane permeability and integrity

Under stress conditions, lysosomal membrane permeabilization (LMP) or full rupture of lysosomes occurs, and the leakage of lysosomal contents into the cytoplasm triggers inflammatory responses and cell death.^26,221,222^ Therefore, defective membrane permeability and integrity may act not only causes of inflammatory diseases but also tools that we can use to treat cancer.^26,223^ Unlike other organelles, lysosomes lack antioxidant enzymes such as superoxide dismutase, which makes their membrane more vulnerable to the damage of ROS and the hydroxyl radicals they produce.^24,224^ Although ROS act as the byproduct of traditional chemotherapies in most cases, they can also be induced intentionally in lysosomes by photodynamic therapy or iron regulation.^225,226^ For example, sequestering iron in lysosomes with ironomycin (AM5) or enhancing the lysosomal degradation of ferritin and the release of iron by artemisinin compounds can evoke the ROS generation and LMP in cancer cells.^227–229^ Direct disrupting LAMP2, the constitutive protein of lysosomal membrane, with mycotoxin enniatin B1 may also be a good strategy to induce LMP.^230^

Furthermore, targeting acid sphingomyelinase (ASM) and its supporter, heat shock protein 70 (Hsp70), can induce LMP by causing sphingomyelin accumulation.^26,231,232^ Direct inhibitors of ASM such as zoledronic acid and Riccardin D-N, as well as functional inhibitors such as cationic amphiphilic drugs (CADs), all show highly efficient ASM inhibition and LMP induction.^26,233,234^ CADs are a wide group of chemicals that can permeate lysosomal membranes and accumulate within lysosomes after protonation, and antimalarials, antidepressants, antihistamines all fall into the CAD category.^24,26,235,236^ In addition to siramesine, CADs like terfenadine and amitriptyline have also been acknowledged to have a great inhibition of ASM and induce LMP in targeted cells (shown in Fig. 4 and Supplementary Table S1).^235,237,238^ The most significant advantages of this category of drugs are the safety and accessibility established by their long-term clinical use.^239^ HSP70 inhibitors such as 2-Phenylethynesulfonamide (PES),^240,241^ quercetin,^242^ triptolide,^242^ and etoposide^243^ show great performance in HSP70 inhibition and LMP induction, but none of them can specifically target lysosomal HSP70.^26,231,240^ Therefore, although these HSP70 inhibitors are of great significance for inducing LMP and subsequent cell death, they may also cause serious adverse reactions because of the simultaneous inhibition of cytoplasmic and membrane HSP70.^231^

Cells have developed numerous defensive mechanisms against lysosomal rupture and subsequent inflammatory responses and cell death.^221,244,245^ It is now generally believed that limited permeabilization of the lysosomal membrane can be repaired through the endosomal sorting complex required for transport (ESCRT) machinery, while badly damaged lysosomes can be engulfed and cleared through the lysophagy machinery, a selective autophagy process triggered by the ubiquitination of lysosomal proteins.^221,246,247^ Three subcomplexes with different functions (ESCRT-I, -II, and -III) have been identified to be involved in the ESCRT mechanism, and the recruitment and translocation of their components have been found heavily dependent on calcium (Ca^2+^) outflowing from lysosomes.^244,245,248,249^ The ubiquitination required for lysophagy is induced the exposure of lysosomal glycans, which are sensed by cytosolic lectins or ubiquitination enzymes.^245^ While cytosolic lectins bind the autophagy receptor NDP52 (nuclear dot protein 52 kDa) and recruit autophagic membranes, ubiquitination enzymes such as ubiquitin conjugating enzyme E2 Q family like 1 (UBE2QL1) and F-box protein 27 (FBXO27) directly mediate the ubiquitination of damaged lysosomal proteins.^245,250–253^ Recently, Gupta et al.^254^ used proteomic-based organelle profiling and identified the selective and high enrichment of myoferlin (MYOF) on the lysosomal membranes of pancreatic cancer cells.^254^ They suggested that MYOF provided early-acting protection against membrane damage by stabilizing the lipid bilayer or promoting the fusion of lysosomes with other vesicles acting as membrane donors rather than through the ESCRT machinery. Lysosomal dysfunction induced by knocking out *MYOF* was demonstrated to impair tumor growth both in vitro and in vivo in this study.^254^ It is possible to modulate the stability and integrity of lysosomal membranes by using these key molecules to intervene in the protective mechanism of lysosomal membranes.

In addition, targeting the microtubule cytoskeleton and inducing mitochondrial membrane permeabilization (MMP) were also found to induce LMP and cell death.^26^ However, the effectiveness of these two approaches seems to be ambiguous because the main cause-and-effect relationship is unclear.^26,255,256^ For example, although there are reports that microtubule regulators, including paclitaxel, vincristine, Deox b7, 4, and BpV (phen), can induce LMP and apoptosis, it is difficult to determine whether cell death is caused by LMP or the disruption of the mitotic spindle, a critical transition in the cell cycle.^26,137,257^ In addition, possible crosstalk between autophagy and LMP offers more options for targeting LMP. Trehalose, an effective autophagy inducer, was found to act by inducing lysosomal enlargement and LMP, while knocking down autophagy protein 5 (*Atg5*) ameliorated IMB-6G-induced LMP and apoptosis.^258,259^

### Target lysosomal calcium signaling

While lysosomes are the main organelles that store intracellular calcium (Ca^2+^), Ca^2+^ mediates the mechanism of lysosomal biogenesis, acidification maintenance, reorganization, and almost all vesicle movements involving lysosomes such as autophagy and endocytosis.^2,260–262^ Growing attention has been paid to the role of lysosomes in the development of cancer and neurodegenerative diseases.^262–264^ Among the Ca^2+^ channels that have been verified in the lysosomal membranes of mammalian cells, there are two groups that are good targets because of their specific localization on the membranes of endo-lysosomal system: transient receptor potential mucolipin channels (TRPMLs) and two-pore channels (TPCs).^2,265,266^

TRPMLs (TRPML1-3) are six-transmembrane domain channels encoded respectively by Mucolipin *(MCOLN) 1-3.*^261,265^ TRPML1 is the best-studied channel and is correlated with lysosome biogenesis and various membrane fusion processes, such as lysosome-autophagosome fusion and plasma membrane repair.^32,33,267–270^ However, its role in cancer progression is much more ambiguous due to its heterogeneous expression.^265,268^ Cancers such as bladder urothelial carcinoma, melanoma, and triple-negative breast cancer, have an upregulated expression of TRPML1.^268,271^ However, there are several examples of cancers with low expression of TRPML1 whose viability can be inhibited by TRPML1 agonists, such as non-small-cell lung carcinoma and glioblastoma.^268,271^ The role of TRPML2 in chemokine trafficking and secretion in murine macrophages was identified, and a bioinformatics analysis correlated the gene encoding TRPML3 with the progression, aggressiveness, and prognosis of pancreatic ductal adenocarcinoma.^272,273^ Some inhibitors and activators with less selectivity for these channels have also been acknowledged or synthesized, such as phosphatidyl-(3,5)-bisphosphate (PI(3,5)P2), ML-SA1, MK6-83, and ML2-SA1 (Fig. 4 and Supplementary Table S1).^265,268^ Besides, agents that target PIKfyve, a phosphoinositide kinase phosphorylates PI(3)P to form PI(3,5)P2, showed good performance in inhibiting the malignant phenotype of autophagy-dependent cancer cells, such as apilimod, YM201636, WX8-family.^274–276^

TPCs are voltage-gated ion channels in the endo-lysosomal system that mediate Ca^2+^ signals through the Ca^2+^-mobilizing messenger nicotinic acid adenine dinucleotide phosphate (NAADP).^266^ Ned-19 and tetrandrine work by targeting NAADP and have shown a great performance in reducing the migration and adhesion of cancer cells such as T24, HUH7, and 4T1-Luc.^265,266^ Besides, Ned-19 and its analog Ned-K were also reported to correct morphological defects in lysosomes in PD caused by *LRRK2* mutations.^277^

Although the important role of Ca^2+^ in neurodegenerative diseases has been established, the corresponding abnormalities in calcium channels have not been established in most neurodegenerative diseases.^261,263,264^ Therefore, it is difficult to select an appropriate calcium channel modulator. The unclear causes and effects of the heterogeneous expression in cancer cells and the lack of drugs with specific targeting also make it difficult to target calcium signaling for cancer therapy.

### Target mTOR signaling

Lysosomes serve as platforms for the proper recruitment, assembly, and activation of mTOR signaling elements, and mTOR acts as a nutrient sensor that regulates the degradation activities of lysosomes.^37,63^ Functionally, lysosomes and mTOR form a tightly connected metabolic complex.^37,63^ While factors such as RAGs, Ragulator, and Rheb contribute to the recruitment of mTOR to lysosomes and its activation, the release of galectin-8 as a result of lysosomal injury leads to the dissociation and activity inhibition of mTOR.^4,37,39,40,278^ MTOR signaling, which modulates cell metabolism and proliferation, is frequently activated in cancer, so mTOR inhibitors can be applied to treat cancer.^37,279^ Since the inhibition of mTOR can induce lysosomal biogenesis and autophagy pathways, mTOR inhibitors can also be used in some diseases with impaired autophagy, such as neurodegenerative diseases (shown in Fig. 4 and Supplementary Table S1).^42–46,280^

While rapamycin (sirolimus) and its analogs (temsirolimus, everolimus, and ridaforolimus) mainly inhibit mTORC1, catalytic mTOR inhibitors (AZD2014, CC-223, TAK-228) inhibit both mTORC1 and mTORC2 through suppressing the catalytic activity of mTOR (shown in Fig. 4 and Supplementary Table S1).^38,281^ Since the PI3K-AKT pathway is involved in the activation and function of both mTOR1 and mTORC2, targeting PI3K or AKT can also achieve simultaneous inhibition of mTORC1 and mTORC2.^38,282^ Considerable progress has been made in the development of drugs targeting PI3K and AKT, and many of them show strong anti-tumor activity both in vivo and vitro, such as buparlisib (BKM120), pictilisib (GDC-0941), MK-2206, Ipatasertib (GDC-0068), and Capivasertib.^281–284^ However, it is worth noting that PI3K and AKT regulates multiple metabolic pathways, so the effect of targeting PI3K or AKT may not depend mainly on mTOR.

Selective inhibition of mTORC1 can be achieved by targeting the unique effector nodes responsible for its recruitment and activation, such as Ragulator, Rheb, and Raptor.^38,285–287^ As a guanine nucleotide exchange factor (GEF) for RAG GTPases, Ragulator signals amino acid levels and recruits mTORC1, and knocking out its essential component, p18, or disrupting its interaction with RAGs by overexpressing c17orf59 has been proven to attenuate aberrant mTORC1 activation.^285,286^ Furthermore, a small molecule called NR1 was reported to bind the switch II domain of Rheb and block mTORC1 signaling potently and selectively.^288^ In addition, a member of the MAP kinase (MAPK) subfamily called Nemo-like kinase (NLK) can phosphorylate Raptor, a distinctive component of mTORC1, resulting in the inhibition of the lysosomal localization of mTORC1 and its subsequent activation.^289^

Attention to the role of mTORC2 in cancer progression is emerging, but it has also been proposed that adverse reactions to the long-term application of mTOR inhibitors are the result of simultaneous mTORC2 inhibition.^63,282,290^ Autophagy induction was once thought to be responsible for weakening the tumor-inhibiting effect of mTORC1 inhibitors, but it now allows the use of rapamycin to treat diseases with impaired autophagy.^38,291^

### Emerging potential targeting strategies

New insights into the mechanisms of the initiation and progression of human diseases associated with autophagic or lysosomal dysfunction have spawned several new targeting strategies. Here, we list several targeting strategies that we believe have great potential, but the lack of drugs with high specificity and efficiency curtails the application of most of these strategies.

Since lysosomal dysfunction is closely correlated with weakened immune signals in the cancer immune response, growing attention has been paid to improving the cancer immune response by targeting disturbed lysosomal degradation.^15,16,292^ While CMTM6 was reported to prevent the lysosomal degradation of PD-L1, which contributes to immune escape, Huntingtin-interacting protein 1 related protein (HIP1R) was found to interact with the conserved domain (771-867) of PD-L1 and transmit it to lysosomal degradation.^16,87^ Huanbin et al. designed and constructed a peptide called PD-LYSO that consists of the PD-L1-binding sequence and the lysosome sorting sequence of HIP1R and demonstrated that this peptide accelerated the lysosomal degradation of PD-L1.^87^ Besides, the proteolysis that targets chimeras called P22 has been identified to restored the immune response in an immunosuppressed coculture model of Hep3B/OS-8/hPD-L1 and CD3 T cells by inhibiting PD-L1 and promoting the lysosomal degradation of PD-L1.^293^ Furthermore, an aloperine derivative called SA-49 was found to decrease the expression of PD-L1 in non-small cell lung cancer cells though promoting the biogenesis of lysosomes and melanogenesis-associated transcription factor (MITF)-dependent lysosomal degradation of PD-L1.^294^ The application of SA-49 was proven to enhance the immune response of cocultured T and NK cells to cancer cells and to suppress the growth of Lewis tumor xenografts in C57BL/6 mice.^294^ Since autophagy was observed to promote the immune evasion of PDAC by degrading MHC-I, scientists tried to combine immune checkpoint blockade (ICB) therapy with autophagy inhibition.^135^ The addition of CQ to anti-PD1 and anti-CTLA-4 antibodies was proven to enhance achieved enhanced anti-tumor immune response in mice with orthotopic tumors.^135^

Targeting TFEB, the transcription factor that regulates the lysosomal–autophagic pathway, has been proved effective for slowing the progression of lysosome-related diseases such as cancer,^295–297^ neurodegenerative diseases,^164–170^ and cardiovascular diseases.^181,298^ Trehalose was found to induce autophagy through promoting the nuclear translocation of TFEB, and this induced autophagy showed protective effects against neurodegenerative diseases,^299^ atherosclerosis,^300^ and cisplatin-induced acute kidney injury.^301^ In addition, a recent review summarized a series of compounds that have been found to regulate the expression or nuclear translocation of TFEB in recent years, such as 3,4-dimethoxychalcone (3,4-DC),^302^ 2-Hydroxypropyl-β-cyclodextrin (HPβCD),^303^ and Digoxin.^298,304^ However, most of these compounds are the modulators of Ca^2+^ signaling or pathways such as mTOR, AKT, and PKC, so it is difficult to determine whether the ultimate effect of these drugs occurs primarily through their effects on TFEB.^298^

Accumulating evidence has acknowledged the contributions of chaperone-mediated autophagy (CMA) to the development of cancers and autoimmune disorders, as well as the protective role of CMA in neurodegenerative diseases.^171–174,305,306^ While knocking down *LAMP2A* was demonstrated to reduce cell proliferation of numerous cancer cells, reduced transcription of heat shock cognate protein 70 (*HSC70*) and *LAMP2A* was reported to aggravate the accumulation of pathological proteins of neurodegenerative diseases, such as α-synuclein, mHTT, and Tau.^172,305,307–309^ In addition, a peptide called P140 was found to inhibit CMA in lupus-prone mice by reducing the expression of both LAMP2A and HSC70 and impairing the refolding properties of HSC70.^310–312^ It has been acknowledged that this inhibition of CMA affected the processing and presentation of autoantigens in B cells and accounts for a decrease in autoreactive T cells.^310,312^

Targeting Rab GTPases (Rabs) is attractive, because they are extensively involved in the biogenesis and function of endo-lysosomal systems.^313,314^ For example, the application of CID1067700, a receptor antagonist of Rab7 GTPase, was identified to inhibit reactive astrogliosis and attenuate brain atrophy of astrocytic injury models though inhibiting excessive transportation of cathepsin B from late endosomes to lysosomes.^315^ However, most Rabs lack specific inhibitors due to their low affinity for nucleotide-based competitive inhibitors and high similarity with each other in structure.^314^ Agents that target their post-translational modification or GTPase–GEF interactions may achieve indirect but efficient inhibition of rabs, such as psoromic acid (PA),^316^ 3-(3-pyridyl)-2-hydroxy-2-phosphonopropanoic acid (3-PEHPC), StRIP3.^314,317^

Although research into the measures and their mechanisms for targeting lysosomes in cancer is still in the primary stage, continuous progress and increasing interest in lysosome research may result in profound developments in this field.

## Representative clinical trials of the targeting strategies

The encouraging results of lysosome-related preclinical studies have aroused growing interest in the clinical transformation and application of targeting lysosomes in cancer. In fact, most of the targeting strategies and corresponding drugs that we summarized above have been tested or are being tested in clinical trials.

### In malignancies

The clinical response to these targeting strategies varies greatly depending on the cancer type, cancer stage, drug type, combination efficacy, the expression of some cancer markers, and the lysosome profile that may have been previously overlooked (Table 2).

**Table 2:** Representative clinical trials of strategies that target lysosomes in malignancies

Drug	Category	Tumor type	Intervention	Phase	Clinical response	Serious adverse events	NCT number	Ref.
CQ	The inhibitor of lysosomal acidification and blocker of the fusion of autophagosomes with lysosomes	Glioblastoma multiforme	RT + TMZ + /− CQ 150 mg/day	III	• Median survival after surgery: 24 months (controls: 11 months).	• Only observed grade 0–1 myelosuppression.	NCT00224978	^323^
CQ		Brain metastases	Whole-brain irradiation + 150 mg CQ 4 weeks or placebo	II	• PFS of brain metastasis at 1 year: 83.9% (control: 55.1%); • ORR: 54% (controls: 55%); • Median OS: 10.2 months (controls: 7.42 months).	• No grade 4 or 5 AEs were observed in either arm; • No significant differences in toxicity between the arms.	NCT01894633	^319^
HCQ		Glioblastoma multiforme	Phase 1: RT + TMZ + /− HCQ 200/400/600/800 mg/day; Phase 2: RT + TMZ + /− HCQ 600 mg/day.	I/II	• No improvements in OS (Median survival: 15.6 months).	• Grade 3 AEs: myelosuppression, rash maculopapular, anemia, elevated ALT; • Grade 4 AEs: myelosuppression.	NCT00486603	^324^
HCQ		Pancreatic Cancer	Preoperative Gemcitabine (1500 mg/m^2^) + HCQ 200, 400, 600, 800, 1000, 1200 mg/day	I/II	• Median OS: 34.8 months (95% CI: (11.57 months, not reached)); • DFS: Patients who had more than 51% increase in LC3-II had improvement in DFS (15.03 vs. 6.9 months); • 61% had a decrease in CA19-9; • R0 resection rate: 77% (prior series: 34%).	• Grade 3 AEs: neutropenia (9%), and the incidence of lymphopenia, hyponatremia, elevated AST, rash, ileus, hypoalbuminemia, hyperbilirubinemia was 3%; • No Grade 4/5 events related to treatment.	NCT01128296	^322^
HCQ		Pancreatic Cancer	Gemcitabine + Abraxane + /− HCQ 600 mg twice daily	II	• OS at 12 months: 41% (controls: 49%); • Median OS: 11.1 months (controls: 12.1 months); • Median PFS: 5.7 months (controls: 6.4 months); • ORR: 38.2% (controls: 12.1 months).	• Neutropenia: 42.6% vs. 22.6%; • Anemia: 3.7% vs. 17.0%; • Fatigue: 7.4% vs. 0; • Nausea: 9.3% vs. 0; • Peripheral neuropathy: 13.0% vs. 5.7%; • Visual changes: 5.6% vs. 0; • Neuropsychiatric symptoms: 5.6% vs. 0.	NCT01506973	^320^
HCQ		Pancreatic Cancer	Nab-paclitaxel and gemcitabine + /− HCQ (1200 mg, 600 mg twice daily)	II	• Median OS: 36 months (controls: 32 months); • Median RFS: 16.6 months (controls: 13.5 months); • Improved serum CA 19-9, immune infiltrate, and pathologic response in the tumor specimen.	• No differences in serious AEs between arms.	NCT01128296	^321^
HCQ		Metastatic pancreatic cancer	400/600 mg HCQ twice a day	II	• Median PFS: 46.5 days; • OS: 69.0 days.	• Treatment-related grade 3/4 AEs were lymphopenia (*n* = 1) and elevated ALT (*n* = 1).	NCT01273805	^318^
Temsirolimus	MTOR inhibitor (rapamycin and its analogs)	Advanced renal-cell carcinoma	**• Group 1:** interferon alfa 3 million U (with an increase to 18 million U) subcutaneously three times a week; **• Group 2:** temsirolimus 25 mg/w intravenously; **• Group 3:** temsirolimus 15 mg/w intravenously + interferon alfa 6 million U three times a week.	III	• Patients in group2 had longer OS (hazard ratio for death, 0.73; 95% confidence interval [CI], 0.58 to 0.92; *p* = 0.008) and PFS (*p* < 0.001) than that of patients in group 1; • There was no significant difference in OS between group 1 and group 3; • The median OS of these three groups are respectively: 7.3, 10.9, and 8.4 months; • The median PFS of these three groups are respectively: 3.1, 5.5, and 4.7 months.	• Percentage of patients with grade 3 or 4 AEs in these three groups are respectively: 78% (*p* = 0.02), 67%, 87% (*p* = 0.02); • Percentage of patients with grade 3 or 4 asthenia in these three groups are respectively: 26% (*p* < 0.001),11%, 28% (*p* < 0.001).	NCT00065468	^328^
Temsirolimus		Recurrent or metastatic endometrial cancer	Temsirolimus 25 mg/w intravenously in 4-week cycles	II	• **In the chemotherapy-naive group:** 14% (4/29) patients were confirmed partial response, 69% (20/29) patients had stable disease as best response, 18% (5/29) patients had progressive disease. • **In the chemotherapy-treated group:** 4% (1/25) patients were confirmed partial response, 48% (12/25) patients had stable disease as best response, 48% (12/25) patients had progressive disease.	• Common grade 3 or grade 4 AEs: fatigue, diarrhea, pneumonitis, and nausea.	NCT01198184	^329^
Everolimus		Advanced gastric cancer	Everolimus 10 mg/day or matching placebo	III	• Median OS: 5.4 months (controls: 4.3 months) (hazard ratio, 0.90; 95% CI, 0.75 to 1.08; *p* = 0.124); • Median PFS: 1.7 months (controls: 1.4 months) (hazard ratio, 0.66; 95% CI, 0.56 to 0.78); • ORR: 4.5% (controls: 2.1%).	• Common grade 3/4 adverse events included anemia, decreased appetite, and fatigue.	NCT00879333	^330^
Vistusertib (AZD2014)	Catalytic mTOR inhibitor	Refractory metastatic renal-cell carcinoma	**Group 1:** 50 mg AZD2014 twice daily; **Group 2:** everolimus 10 mg/day.	II	• PFS for AZD2014 and everolimus are respectively: 1.8 months and 4.6 months (hazard ratio: 2.8 [95% confidence interval (CI), 1.2–6.5]; *p* = 0.01); • Progression of disease as the best response to therapy was 69% for AZD2014 and 13% for everolimus (*p* < 0.001); • Pharmacokinetics analysis: the concentrations of AZD2014 were compatible with the therapeutic range.	• Grade 3–4 AEs occurred in 35% of AZD2014 and 48% of everolimus patients (*p* = 0.3).	Not applicable	^331^
CC-223		Non-pancreatic neuroendocrine tumors	Oral administration of CC-223 45 mg/day in 28-day cycles with a subsequent cohort starting at 30 mg/day	I /II	• The objective response rate (complete response + partial response) was 7.3% (95% CI 1.5–19.9%); • The disease control rate (complete response + partial response + stable disease) was 90.2% (95% CI 76.9–97.3%); • Median PFS: 19.5 months (95% CI 10.4–28.5 months).	• Most frequent grade ≥3 toxicities were diarrhea (38%), fatigue (21%), and stomatitis (11%).	NCT01177397	^332^
TAK-228 (MLN0128)		Metastatic castration-resistant prostate cancer	TAK-228 4 mg/day	II	• 8 of 9 patients (89%) discontinued treatment before the scheduled 6-month trial endpoint; • All patients had a rise in PSA on treatment, with a median 159% increase from baseline (range: 12–620%); • No patient had a decrease in circulating tumor cell count.	• The most common serious adverse events were grade 3 dyspnea and maculopapular rash.	NCT02091531	^333^
MK-2206	Allosteric pan-AKT inhibitor	Advanced solid tumors	60 mg MK-2206 on alternate days or receiving MK-2206 at 90, 135, 150, 200, 250, and 300 mg/w	I	• The MTD of weekly medication was 200 mg; • Significant decrease of pSer473 AKT signal was observed in two groups: 50.0% vs. 50.1%.	• 3 patients experience grade 3 rash at MTD; • No treatment-related grade 4–5 AEs were observed.	NCT00670488	^335^
AZD5363	ATP-competitive pan-AKT inhibitor	ER ( + ), HER2 (-) breast cancer	Fulvestrant + AZD5363 400 mg twice daily or placebo	II	• Rate of PFS events: 71% (controls: 89%); • Median PFS: 10.3 months (controls: 4.8 months); • Objective response rate: 29% (controls: 8%); • Median OS: 26.0 months (controls: 20.0 months); • Median duration: 9·2 months (controls: 4.6 months).	• The most common grade 3–4 AEs: hypertension (32% vs. 24%), diarrhea (14% vs. 4%), rash (20% vs. 0), infection (6% vs. 3%), and fatigue (1% vs. 4%).	NCT01992952	^337^
Temsirolimus + HCQ	mTOR inhibitor+ autophagy inhibitor	Advanced solid tumors and melanoma	Temsirolimu 25 mg + HCQ 200/400/800/1200 mg/day	I	• No responses were observed; • 14/19 (74%) patients with melanoma achieved stable disease; • Median PFS of melanoma patients treated with temsirolimus combined with HCQ 1200 mg/day: 3.5 months.	• Grade 3 or 4 toxicity: anorexia (7%), fatigue (7%), and nausea (7%).	NCT00909831	^340^
Everolimus + HCQ	Dual PI3K-mTOR inhibitor + autophagy inhibitor	Renal-cell carcinoma	Everolimus 10 mg/day + HCQ 400/600 mg twice daily (beginning 1 week later)	I/ II	• Rate of disease control: 67%; • Rate of partial response: 6%; • Rate of PFS ≥ 6 months: 45%; • Median PFS: 6.3 months.	• Grade 3–4 AEs: fatigue (8%), anemia (8%), nausea (5%), anorexia (5%), elevated triglycerides (5%), hyperglycemia (5%), neutropenia (5%).	NCT01510119	^341^
Amitriptyline	Antidepressant	Cancer patients with CIPN	**Group 1**: ketamine + amitriptyline 4 g twice daily (cream); **Group 2**: Placebo.	III	• No decrease of CIPN symptoms was made in cancer survivors.	• Most common AEs including musculoskeletal, gastrointestinal, skin, neurological, and fatigue were similar between arms.	NCT00471445	^342^
MKT-077	Hsp70 inhibitor	Advanced solid cancers	30, 40, and 50 mg/m^2^/day for 18 cycles	I	• The trial was halted because that irreversible renal toxicity was observed in animal studies.	• Reversible nephrotoxicity	Not applicable	^344^
ZA	ASM inhibitor	Breast Cancer	Tamoxifen/anastrozole + /− ZA 4 mg/6 m	III	• DFS: 88.4% (without ZA:85.0%); • Disease recurrences: 111 (without ZA:140); • OS rate: 96.7% (without ZA: 94.5%).	• No safety concerns were evident 5 years after median treatment completion.	NCT00295646	^386^
ZA		Breast Cancer	Letrozole + immediate/delayed ZA 4 mg/6 m	III	• Mean change in lumbar spine BMD: + 4.3% (delayed ZA: −5.4%); • Reduce the risk of DFS events by 34%; • Local recurrence: 0.9% (delayed ZA: 2.3%); • Distant recurrence: 5.5% (delayed ZA: 7.7%).	• Fractures and atrial fibrillation were statistically similar in the immediate and delayed-zoledronate arms; • Renal AEs were similar between treatment arms.	NCT00171340	^345^
Odanacatib	Cathepsin K inhibitor	Breast cancer with bone metastases	**Group 1:** ZA 4 mg at the start of treatment; **Group 2:** odanacatib 5 mg/day for 4 weeks.	II	• Bone resorption: The mean percent change in uNTx values at week 4 was –77% (ZA: –73%).	• Serious AEs in the odanacatib group included febrile neutropenia, ascites, and 2 incidents of progression of bone metastases.	NCT00399802	^348^

*AE* adverse events, *CQ* chloroquine, *HCQ* hydroxychloroquine, *ZA* zoledronic acid, *TMZ* temozolomide, *RT* radiation therapy, *PFS* progression-free survival, *RFS* recurrence-free survival, *DFS* disease-free survival, *ORR* overall response rate, *OS* overall survival, *uNTx* urinary N-telopeptide of type I collagen corrected for creatinine, *CIPN* chemotherapy-induced peripheral neuropathy, *Hsp70* heat shock protein 70, *BMD* bone mineral density, *ER (* *+* *)* estrogen receptor-positive, *HER2* (−) human epidermal growth factor receptor 2 negative, *ALT* alanine aminotransferase, *AST* aspartate aminotransferase, *ASM* acid sphingomyelinase. +/– with or without

With incomparable easy-accessibility and safety as a result of long-term clinical use in antimalarial therapy, CQ and HCQ have been rapidly and widely repurposed as agents targeting lysosome acidification in anticancer clinical trials.^5,200^ However, their effectiveness as single drugs against cancer is very limited in practice, so researchers are now using them as sensitizers in combination with radiotherapy and chemotherapy drugs such as taxane, carboplatin, gemcitabine, temozolomide, and metformin.^318–322^ In a search of the ClinicalTrials.gov website, we found that numerous clinical trials (almost half) used glioblastomas or tumors with brain metastases as cancer targets for CQ, and most of the trials showed improved results, which might indicate the superiority and specificity of CQ in the treatment of cerebral tumors (Table 2).^319,323^ For example, adding CQ to whole-brain irradiation was confirmed to play a great role in the treatment of patients with brain metastases, with a 1-year progression-free survival rate of 83.9% compared with 55.1% in the control group.^319^

However, a trial that combined HCQ with temozolomide and radiotherapy to treat newly diagnosed glioblastoma multiforme ended up with no significant improvement.^324^ Some scholars believed that the inconsistent levels of autophagy inhibition were the main reasons for the unsatisfactory results.^5^ Besides, researchers are trying to use this effective sensitizer in the treatment of pancreatic cancer, a highly lethal cancer that requires high autophagy level to maintain metabolism and resist therapy.^203,320–322^ While adding HCQ to gemcitabine or gemcitabine plus nab-paclitaxel was determined to improve prognosis-related serum biomarker (CA19-9), opinions were divided regarding its ability to promote overall survival (OS) and progression-free survival (PFS) (Table 2).^320–322^ There are two clinical trials that respectively used SQSTM1/p62 and LC3-II as the marker of effective inhibition of autophagy, and one of the trials measured both markers, which showed inconsistent levels of autophagy inhibition.^321,322^ Consequently, the latter trial may not have achieved effective autophagy inhibition, which indicates the lack of a unified standard for autophagy inhibition. In addition, the imbalance in KRAS mutations between the experimental group and the control group may also affect the experimental results.^320^ Although the clinical response to HCQ is not always satisfactory, it remains the most widely used antimalarial drug in clinical anti-tumor research.^5^

Temsirolimus and everolimus are mTOR inhibitors that have been approved by the US FDA for cancer treatment.^325–327^ In the treatment of advanced renal-cell carcinoma, using temsirolimus as a single agent has been identified to achieve longer OS and PFS than interferon monotherapy, and showed no significant difference in OS compared with the combination-therapy of temsirolimus and interferon (Table 2).^328^ This clinical trial data was thought to contribute to the US FDA’s approval of temsirolimus for the treatment of advanced renal-cell carcinoma.^325^ Temsirolimus also showed great efficiency in metastatic endometrial cancer (Table 2).^329^ Although everolimus was approved by the US FDA for the treatment of numerous cancers, it was found to have very limited efficacy when used as a single agent (Table 2).^326,330^ Since the activities of mTORC2 may compensate for the inhibition of mTORC1, a series of inhibitors that simultaneously target mTORC1 and mTORC2 have been developed, such as vistusertib (AZD2014),^331^ CC-223,^332^ and TAK-228 (MLN0128).^333^ Among these drugs, vistusertib is the most clinically used, but it was shown to result in lower OS and PFS improvement than everolimus.^331^ The efficacy of TAK-228 in metastatic castration-resistant prostate cancer was also reported to be limited, and eight of nine patients discontinued the treatment early because of radiographic progression, drug toxicity, or investigator discretion.^333^ CC-223 was proven effective and safe for the treatment of non-pancreatic neuroendocrine tumors, and clinical trials of this drug in other cancers are underway.^332^

Although inhibitors of the PI3K-AKT pathway showed great efficiency in cancer therapy, mTOR signaling is not its only downstream signaling pathway, and the activation status of mTOR signaling was not reported in these clinical trials.^334–339^ Since the inhibition of mTOR always acts as a potent inducer of cytoprotective autophagy that greatly compromises therapeutic effects, researchers have tried to combine mTOR inhibitors with autophagy inhibitors.^283,340,341^ Temsirolimus was tolerable and efficient when combined with HCQ in the treatment of melanoma and multiple advanced solid tumors, and the combination of everolimus with HCQ in patients with renal-cell carcinoma also achieved the primary endpoint without dose-limiting toxicity observed.^340,341^

Inducing LMP directly leads to cell death in preclinical experiments, but the effectiveness of most drugs used for this strategy are not satisfactory. For example, little improvement has been achieved in the application of CADs for cancer therapy, except for antimalarials.^342,343^ Amitriptyline, a tricyclic antidepressant, failed to decrease chemotherapy-induced peripheral neuropathy (CIPN) symptoms in cancer survivors, and a trial of desipramine for the treatment of patients with small cell lung cancer was terminated early because of intolerable doses and a lack of clinical activity.^342,343^ Although mountains of HSP70 inhibitors have been synthesized and identified to be effective in vitro, few HSP70 inhibitors have been tested in clinical trials.^231^ MKT-077 is possibly the only Hsp70 inhibitor that has been tested in clinical trials against cancer currently, but the trial was halted because of irreversible renal toxicity.^344^ ASM inhibitors and agents that target microtubules, the other two classes of LMP inducers, have been much more widely used in clinical studies with good results.^26,239,345,346^ Among them, ZA, paclitaxel, and vincristine have been used as standard treatments for many cancers.^26,239,346^ Nevertheless, It is worth noting that their therapeutic effects in the treatment of cancers are not confined to their direct effects in lysosomes, and their induction of LMP in cancer cells is seldom examined in clinical trials.^26,137,257^

Few cathepsin inhibitors have been used in clinical trials, and only one trial of the cathepsin K inhibitor odanacatib in cancer therapy has been completed.^347^ Although odanacatib achieved bone resorption inhibition comparable to that of ZA in the treatment of patients with bone metastases of breast cancer, there were some limitations in this trial, such as the relatively small sample size and the lack of clinical outcomes as efficacy endpoints.^348^ In a multicenter phase 3 clinical trial of osteoporosis, the long-term use of odanacatib (median follow-up 47.6 months) was found to significantly increase the risk of cardio-cerebrovascular events, especially stroke.^216^ Therefore, the study’s sponsor decided not to develop it as a treatment for osteoporosis (in 2019).^216^ Since then, there have been no clinical trials or applications for clinical trials of odanacatib for cancer treatment.^347^ However, we still believe that this drug has potential for treating cancer bone metastases because patients diagnosed with cancer bone metastases generally have a short survival period and will not take the medication for such long periods.^349^

The understanding of lysosomal calcium channels is still in the primary stage, and there is an urgent need for targeted drugs with high specificity.^14^ The emerging targeting strategies that we summarized above faced run into similar dilemmas. More highly effective drugs and corresponding clinical trials are needed to judge the effectiveness, safety, and feasibility of these targeting strategies.

### In non-malignant diseases

With milder gastrointestinal and skin complications than CQ, HCQ has been more widely used clinically and in clinical trials.^156^ According to the management recommendation of European League Against Rheumatism (EULAR) published in 2019, HCQ is recommended for all patients with SLE.^350^ In a clinical trial, patients with higher blood levels of HCQ (≥1000 ng/ml) were reported to be less likely to develop active SLE.^351^ Nevertheless, both EULAR and American Academy of Ophthalmology recommend that the daily dose of HCQ should not exceed 5 mg/kg actual body weight.^350,352^ Using HCQ as either a single or a combinatorial therapy has been proven to be effective in the treatment of RA, but the addition of HCQ has been found to decrease maximum concentration of methotrexate (MTX) and increase the risk of MTX-induced toxicities (Table 3).^353,354^ In a prospective, multicenter observational study of 4905 RA patients, Mary Wasko et al.^355^ found that using hydroxychloroquine was correlated with a low risk of suffering diabetes. However, in a clinical trial, RA patients who received HCQ 6.5 mg/kg daily for 8 weeks showed no difference in insulin resistance and a slight improvement in lipid levels (total cholesterol and low-density lipoprotein) compared with controls.^356^ A possible reason for the different conclusions of the trial may be the short observation time, and a longer trial time may make the improvement more obvious. Since HCQ may improve lipid metabolism in human body, some scholars believe that the use of HCQ in RA patients can reduce the frequency of cardiovascular events.^357–360^

**Table 3:** Representative clinical trials of strategies that target lysosomes in non-malignant diseases

Drug	Category	Disease	Intervention	Phase	Clinical response	Adverse events	NCT number	Ref.
HCQ	The inhibitor of lysosomal acidification and block the fusion of autophagosomes with lysosomes	SLE	• **Group 1:** no daily dose change of HCQ; • **Group 2:** increased HCQ dose to achieve the target.	IV	• Active SLE was less prevalent in patients with higher blood HCQ levels; • SLE flare rates were similar in the two groups (25% vs. 27.6%, *p* = 0.7); • Patients at the therapeutic target had fewer flares than those with a low blood level of HCQ (20.5% vs. 35.1%, *p* = 0.12).	• Rate of AEs in two groups: 20.2%vs.26.4%; • Common AEs: nausea, vomit, diarrhea, pruritus, blurred vision.	NCT00413361	^351^
		Cutaneous lupus erythematosus	• **Period 1** (16 weeks): HCQ 200~400 mg/day or placebo; • **Period 2:** a same dose of HCQ.	III	• Mean change of CLASI score at week 16 was not significantly different (−4.6 vs. −3.2, *p* = 0.197); • Rate of patients showed “improved and remarkably improved” at week 16: 59.4% vs. 30.4% (*p* = 0.029).	• AEs related to HCQ: cellulitis, drug eruption, hepatic dysfunction, and Stevens-Johnson syndrome.	NCT01551069	^387^
		RA	• **Period 1** (8 weeks): HCQ (6.5 mg/kg/day) or placebo for the first 8 weeks; • **Period 2** (8 weeks): crossover to the other arm.	III	• Mean ± SD ISI increased at 8 weeks: 0.4 ± 2.9 (placebo: 0.14 ± 3.1) (adjusted *p* = 0.785); • Mean ± SD HOMA-IR decreased at 8 weeks: 0.3 ± 1.5 (placebo: 0.42 ± 1.4) (adjusted *p* = 0.308); • Small decreases in total cholesterol and low-density lipoprotein cholesterol were observed during the HCQ treatment periods.	• Not reported	NCT01132118	^356^
		People at high risk of diabetes	HCQ 400 mg/day vs. placebo	IV	• Positive change in insulin sensitivity (mean ± SEM) : +20.0% ± 7.1% (control: −18.4% ± 7.9%, *p* < 0.01); • Improvement in beta cell function: +45.4% ± 12.3% (control: −19.7% ± 13.6%).	• No serious or unexpected adverse effects	NCT01326533	^388^
P140 peptide (IPP-201101)	CMA inhitor (inhibit CMA by binding to HSC70)	SLE	• **Group 1:** 200 μg Ipp-201101 was injected subcutaneously (SC) every 2 weeks, 3 times; • **Group 2:** 1000 μg Ipp-201101 was injected subcutaneously (SC) every 2 weeks, 3 times.	II	Patients with decreased IgG anti-dsDNA antibody levels (≥20%): 7 patients in group1 and 1 patient in group2 (the total number of each group was 10); • Proportion of patients achieving a reduction of at least 4 points in the SLEDAI score: 60% vs. 44%.	• No clinical or biological adverse effects were observed in the individuals.	Not applicable	^361^
Lupuzor (P140 peptide, IPP-201101)		SLE	• **Group 1:** lupuzor (200 μg) subcutaneously every 4 weeks; • **Group 2:** lupuzor (200 μg) subcutaneously every 2 weeks; • **Group 3:** placebo.	II	• In the intention-to-treat overall population, rate of patients achieved SRI response at week 12 are respectively 53.1% (*p* = 0.048), 45.1% (*p* = 0.18), and 36.2%; • In patients with SLEDAI score ≥6 at week 0, the rate of patients who achieved SRI response at week 12 are respectively 61.9% (*p* = 0.016), 48.0% (*p* = 0.18), and 38.6%; • Efficacy according to the interim analysis (group 1 compared with placebo): at week 12, 67.6% vs 41.5% (*p* < 0.025); at week 24, 84.2% vs 45.8% (*p* < 0.025).	• Incidence of AEs through week 24 was similar among the treatment groups; • The most common AE: injection-site erythema. • Serious AEs: Pneumonia (one patient in group 1 and two patients in group 3); Herpes viral pneumonia (one patient in group 2); Soft-tissue infection (one patient in group 1); Diverticulitis (one patient in group 3); Gastritis (one patient in group 1).	Not applicable	^363^
Lupuzor (P140 peptide, IPP-201101)		SLE	• **Group 1:** standard of care + 200 μg subcutaneously every 4 weeks; • **Group 2:** standard of care + placebo.	III	• Percentage of patient responder (SRI at week 52): 52.5% vs. 44.6%, *p* = 0.2631; • Percentage of patient responder (anti-dsDNA at week 52): 61.5% vs. 47.3%.	• Patients with serious AEs: Group 1: 13/101 (12.87%) Group 2: 16/101 (15.84%).	NCT02504645	^362^
Sirolimus	mTORC1 inhibitor	Active SLE	The initial dose of sirolimus is 2 mg/day, and then adjusted according to the situation.	I/II	• Mean SLEDAI score at week 12: 4.8 (at enrollment: 10.2, *p* < 0.001); • Mean total BILAG index score at week 12: 17.4 (at enrollment: 28.4, *p* < 0.001); • Mean daily dose of prednisone required to control disease activity at week 12: 7.2 mg (at enrollment: 23.7 mg, *p* < 0.001); • Expanded CD4 + CD25 + FoxP3+ regulatory T cells and CD8 + memory T-cell populations and decreased IL-4 and IL-17 production by CD4 + and CD4 CD8 double-negative T cells after 12 months.	• HDL-cholesterol, neutrophil counts, and hemoglobin were moderately reduced within a safe range.	NCT00779194	^367^
		Active RA	Conventional therapy with or without sirolimus (0.5 mg on alternate days) for 24 weeks	I/II	• Significant reduction in disease activity indicators including DAS28, ESR, and the number of tender joints and swollen joints (*p* < 0.001); • Higher level of Tregs as compared with those with conventional therapy alone (*p* < 0.05).	• No difference in blood routine, and liver and renal functions between the two groups (*p* > 0.05).	Not applicable (Registered at the Chinese Clinical Trial Registry)	^366^
RO5459072	Cathepsin S inhibitor	Primary Sjogren’s Syndrome	• **Group 1:** matching placebo; • **Group 2:** RO5459072 100 mg orally, 2 times a day, for up to 12 weeks.	II	• Percentage of participants with a clinically relevant decrease in ESSDAI score: 37.8% vs. 42.1% (*p* = 0.7955); • Percentage of participants with a clinically relevant decrease in ESSPRI score: 56.8% vs. 57.95 (*p* = 0.9877); • Change from baseline in ESSDAI score: –3.06 vs. –3.25 (*p* = 0.8905); • Change from baseline in ESSPRI score: –1.35 vs. –1.51 (*p* = 0.6077).	• Percentage of serious AEs: 5.41% (2/37) vs. 2.63% (1/38); • Percentage of other AEs: 51.35% (19/37) vs. 65.79% (25/38); • Common AEs: gastrointestinal disorders, skin and subcutaneous tissue disorders, nervous system disorders, infections, and infestations.	NCT02701985	^370^
Odanacatib (MK-0822)	Cathepsin K inhibitor	Osteoporosis	• **Group 1:** 50 mg/w odanacatib orally + 5600 IU/w vitamin D3 orally + 1200–1600 mg/day calcium intake; • **Group 2:** matching placebo + 5600 IU/w vitamin D3 orally + 1200–1600 mg/day calcium intake.	III	**In LOFT (median follow-up 36.5 months):** • Cumulative incidence of radiographic vertebral fractures: 3.7% vs. 7.8%, HR 0.46, 95% CI 0.40–0.53; • Cumulative incidence of hip fractures: 0.8% vs. 1.6%, HR 0.53, 95% CI 0.39–0.71; • Cumulative incidence of non-vertebral fractures 5.1% vs. 6.7%, HR 0.77, 0.68–0.87. (All *p* < 0.0001) **LOFT plus LOFT Extension (median follow-up 47.6 months):** • Cumulative incidence of radiographic vertebral fractures: 4.9% vs. 9.6%, HR 0.48, 95% CI 0.42–0.55; • Cumulative incidence of hip fractures: 1.1% vs. 2.0%, HR 0.52, 95% CI 0.40–0.67; • Cumulative incidence of non-vertebral fractures 6.4% vs. 8.4%, HR 0.74, 95% CI 0.66–0.83. (All *p* < 0.0001)	**In LOFT:** • The rate of composite cardiovascular endpoint: 3.4% vs. 3.1% (HR 1.12, 95% CI 0.95–1.34; *p* = 0.18); • The rate of new-onset atrial fibrillation or flutter: 1.4% vs. 1.2% (HR 1.18, 95% CI 0.90–1.55; *p* = 0.24); • The rate of stroke: 1.7% vs. 1.3% (HR 1.32, 95% CI 1.02–1.70; *p* = 0.034). **LOFT plus LOFT Extension:** • The rate of composite cardiovascular endpoint: 5.0% vs. 4.3% (HR 1.17, 1.02–1.36; *p* = 0.029); • The rate of stroke: 2.3% vs. 1.7% (HR 1.37, 1.10–1.71; *p* = 0.0051).	NCT00529373	^216^

*HCQ* hydroxychloroquine, *AE* adverse event, *SLE* systemic lupus erythematosus, *RA* rheumatoid arthritis, *CLASI* cutaneous lupus erythematosus disease area and severity index, *ISI* insulin sensitivity index, *HOMA-IR* homeostatic model assessment for insulin resistance, *SD* standard deviation, *SEM* standard error of mean, *CMA* chaperon-mediated autophagy, *SRI* SLE Responder Index, *SLEDAI* Systemic Lupus Erythematosus Disease Activity Index, *BILAG* British Isles Lupus Assessment Group, *EULAR* European League Against Rheumatism, ESSDAI EULAR Sjogren’s Syndrome Disease Activity Index, *ESSPRI* EULAR Sjogren’s Syndrome Patient-Reported Index Score, *LOFT* the long-term odanacatib fracture trial, *HR* hazard ratio, vs. versus, mg/d mg once a day, mg/w mg once a week^389–414^

P140, a phosphopetide that can inhibit CMA, was found to decrease the levels of IgG anti-dsDNA antibody in patients with SLE and effectively improve their SLE disease activity index (SLEDAI) score with no adverse effects.^361^ Intriguingly, in another phase 2 clinical trial of patients with SLE, the subcutaneous injection of 200 μg of P140 every 4 weeks was found to achieve a better SLE responder index (SRI) response at 12 weeks than the same dose given every 2 weeks or a placebo control.^362^ However, a phase III clinical trial reported that the SRI response rate of patients treated with P140 (200 μg every 4 weeks) showed no significant difference (52.5% vs. 44.6%, *p* = 0.2631) from that of the control group at week 52.^362^ Therefore, P140 is safe and well tolerated for the treatment of SLE, but its efficacy in achieving long-term remission and control of SLE may require further experimental evidence.^361–363^

Growing attention has been paid to the role of T cells in autoimmune diseases.^364,365^ As described above, mTOR signaling is involved in modulating the differentiation of T cells, so a number of clinical trials have been conducted to test the efficacy of its inhibitor in the treatment of autoimmune diseases.^64,65,366,367^ Low-dose sirolimus, an inhibitor that mainly acts on mTOC1, has been found to selectively upregulate Tregs and achieve better control of the disease activity of RA (Table 3).^366^ In patients with SLE, sirolimus was also effective for controlling disease activity and correcting pro-inflammatory T-cell lineage specification (Table 3).^367^ However, there are two issues that attract our attention. First, the use of mTOR inhibitors may induce autophagy, which is always activated in immune cells in autoimmune diseases and is closely related to disease development.^283,368^ Further studies are needed to determine autophagy activation at these doses and the necessity of combing sirolimus with autophagy inhibitors.^283,368^ Second, while the upregulation of nonselective IgG or autoantibodies is common in SLE, the promotion of Th2 cell differentiation as a result of mTORC1 inhibition may facilitate humoral immunity and aggravate the accumulation of autoimmune complexes.^64,141,369^

As we explained previously, mTORC1 inhibitors can also be used as autophagy inducers to treat neurodegenerative diseases. Several clinical trials of mTOR inhibitors for the treatment of PD and AD are actively recruiting, such as NCT04629495, NCT04200911, and NCT04127578. Trehalose, which induces autophagy via TFEB activation, are also testing in the clinical trial of AD (NCT04663854). These drugs may be valuable candidates for the treatment of neurodegenerative diseases.

Numerous cathepsin inhibitors have been developed, but few of them have been tested in clinical trials. A trial assessing the efficacy of RO5459072 in patients with primary SS was completed, but no significant improvement in the EULAR Sjogren’s Syndrome Disease Activity Index (ESSDAI) score was observed in the RO5459072 group.^370^ Other targeting strategies have only come to the forefront in recent years, and their corresponding drugs are still in development or undergoing preclinical testing.

## Conclusion and perspectives

Although targeted lysosomes show great promise for treating human diseases such as malignancies, autoimmune diseases, neurodegenerative diseases and cardiovascular diseases, there are still many questions waiting to be answered. A key question that needs to be answered first is how to selectively target lysosomes, especially lysosomes in pathological cells. CADs, including CQ, have good lysosomal aggregation characteristics, but they may result to indiscriminate inhibition or deletion of all lysosomal function.^24^ Although lysosomal changes in diseases are the foundation of our targeting strategies, there is no clinical evidence that these changes allow currently available therapies to strike fatal blows to pathological cells far more often than to normal cells.^2,14^ Luckily, metabolic glycoengineering of unnatural sugars provides a powerful tool for selectively labeling cancer cells, and antibody-drug conjugates restrict the systemic delivery of antineoplastic agents to cells that express certain antigens.^371,372^ In addition, nanoscale drug delivery systems, such as cathepsin-sensitive drug delivery systems, possess the unique ability to penetrate cell barriers and locate in certain organelles such as lysosomes.^14,373^ These emerging approaches offer the possibility of specifically targeting lysosomes, especially lysosomes in pathological cells.

Another question arises from the heterogeneity and complexity of lysosomes and their associated pathways. Several commonly targeted molecules or pathways exert dual effects on cancer progression and adapt with time, and the broad-spectrum and complete suppression of these molecules and pathways may lead to unpredictable and irreparable side effects.^2,14,235,374^ Therefore, it is necessary to develop precise personalized treatment regimens and monitor the functional status of lysosomes in cancer patients in real-time. The expression of some specific molecules can be used to evaluate the activation of the pathways, and the introduction of microfluidic single-cell analysis technology is expected to achieve lysosomal level accuracy in the future.^14,101,246^ However, these methods have not currently achieved the real-time monitoring of lysosomal number and function in individuals. In addition, tremendous work is needed to develop uniform and implementable guidelines for standardizing research and diagnosis.^14,321^ For instance, a trial that evaluated both SQSTM1/p62 and LC3-II found inconsistent levels of autophagy inhibition, making it difficult to determine whether autophagy was effectively suppressed.^321^

The issue of drug specificity and efficacy also requires attention. The structural and functional similarities among molecules in the same family make it difficult to target specific molecules, and the complementary effects of these molecules may weaken the targeting effect.^207,314^ For example, when cathepsin L is inhibited, cathepsin B activity increases compensatively and partially offsets the effect of cathepsin L inhibition.^207^ However, if both are inhibited simultaneously, other related pathways of cathepsin B will be affected.^207^ Future research needs to not only develop targeting agents with high selectivity but also clarify the necessity and extent of the inhibition of compensatory molecules. In addition, many targeting agents have limited therapeutic effects as single agents and need to be used in combination.^318–322,326,330^ Although combination-therapy can improve the efficacy of these compounds, it may also increase the side effects of the drugs and aggravate the metabolic burden on patients’ liver and kidneys.^97^ Therefore, there is an urgent need to develop more potent drugs and identify the tumors that are sensitive to them.

In conclusion, there are still many difficulties and challenges to be overcome, but they are not completely unsolvable considering the improvement of lysosome-targeted drugs and the optimization of research instruments and methods. Lysosomes are important regulators of cell and organismal homeostasis that mediate energy metabolism, cell proliferation and differentiation, immunity, and cell death. The lysosomes of a variety of disease cells have been found to undergo some lysosomal changes and dysfunction that have a profound effect on disease progression. Lysosomal acidification, lysosomal cathepsins, lysosomal membrane permeability and integrity, lysosomal calcium signaling, mTOR signaling, lysosomal degradation of immune signals, TFEB, noncanonical autophagy, and vesicle movement are all promising targets for lysosomes, and some of these targeted drugs have been tested clinically effective and safe. Therefore, targeting lysosomes in human disease is a feasible, effective, and safe targeted strategy, and we can look forward to developing it as an excellent therapeutic intervention.

## Supplementary information

Supplementary Table S1. Available strategies for targeting lysosomes in human disease and their corresponding drugs

## Data Availability

All data generated or analyzed during this study are included in this published article.
