# Supplementary material for: Targeting lysosomes in human disease: from basic research to clinical applications
Source: Signal Transduct Target Ther. 2021 Nov 8;6:379. doi: 10.1038/s41392-021-00778-y (PMC8572923; doi:10.1038/s41392-021-00778-y)
Supplement: Supplementary file 1 — Supplementary Table S1. Available strategies for targeting lysosomes in human disease and their corresponding drugs [file 41392_2021_778_MOESM1_ESM.docx]

**Supplementary Table S1. Available strategies for targeting lysosomes in human disease and their corresponding drugs.**

| **Targeting strategies** | | **Targeting agent** | **Mode of action** | **Cell type or animal model** | **Clinical trial phase** | **Ref** |
| --- | --- | --- | --- | --- | --- | --- |
| **Target lysosomal acidification** | **Inhibitor** | Bafilomycin A1 | •Inhibit V-ATPase-dependent acidification;  •Inhibit Ca-P60A/SERCA-dependent autophagosome-lysosome fusion | Multiple cell types | Preclinical | ^194^ |
|  |  | Concanamycin | •Inhibit V-ATPase through preventing the rotation of ATP6V0C multimer | Multiple cell types | Preclinical | ^189^ |
|  |  | INDOL0 | •Inhibit V-ATPase through binding its V0 subunit c | Endogenous membranes isolated from hepatopancreas of Nephrops norvegicus | Preclinical | ^192^ |
|  |  | Archazolid A | •Inhibit V-ATPase through binding its V0 subunit c | **Cell type:** HMLE, SKBR3 (breast cancer), Hela cells (cervical cancer).  **Animal model:**  BALB/cByJRj mice inoculated with 1 * 10^5^ 4T1-Luc cells via the tail vein | Preclinical | ^191,389^ |
|  |  | Salicylihalamide A; | •Inhibit V-ATPase through impeding the V0 domain and redistributing cytosolic V1 | The clathrin-coated vesicle V-ATPase isolated from bovine brain | Preclinical | ^195^ |
|  |  | VoPQ | •Inhibit V-ATPase through binding its Vo subcomplex | BY4742, BJ3505, DKY6281 (yeast cells) | Preclinical | ^390-392^ |
|  |  | CQ | •Deacidify lysosomes；  •Block the fusion of autophagosomes with lysosomes | Multiple cell types | I, II, III | ^5,97^ |
|  |  | HCQ | •Deacidify lysosomes；  •Block the fusion of autophagosomes with lysosomes | Multiple cell types | I, II | ^5,26^ |
|  |  | QN | •Deacidify lysosomes | Multiple cell types | I, II | ^133^ |
|  |  | Lys05 | •Deacidify lysosomes;  •Impair mTORC1 and lysosomal catabolism by targeting PPT1 | **Cell type:** LN229 (glioma), 1205Lu (melanoma), HT-29 (colon), and c8161 (melanoma) cells.  **Animal model:** *Nu/Nu* nude mice bearing c8161 xenografts, *Nu/Nu* nude mice bearing 1205Lu xenografts, and *Nu/Nu* nude mice bearing HT-29 colon cancer xenografts. | Preclinical | ^203,393^ |
|  |  | DQ661 | •Deacidify lysosomes;  •Impair mTORC1 and lysosomal catabolism by targeting PPT1 | **Cell type:** melanoma cell lines: A375P, 451Lu, 1205Lu, C8161, WM1361A, and WM3918; Colon cancer cell line: HT-29; Pancreatic cancer cell lines: CAPAN1 and PANC-1.  **Animal model:** NSG mice bearing 1205Lu melanoma xenografts, NSG mice bearing HT-29 xenografts, and C57BL/6 mice bearing G43 (gemcitabine-resisted cell) xenografts. | Preclinical | ^203^ |
|  |  | DC661 | •Deacidify lysosomes;  •Impair mTORC1 and lysosomal catabolism by targeting PPT1. | **Cell type:** melanoma cell lines: A375P, WM3918, WM983B; Colon cancer cell line: HT-29; Pancreatic cancer cell line: PANC-1.  **Animal model:** NSG mice bearing HT-29 xenografts. | Preclinical | ^204^ |
|  | **Inducer** | PLGA-aNP | •Acidic nanoparticles act on lysosomal PH | **Cell type:** mutant ATP13A2, GBA and XMEA fibroblasts (Parkinson's disease models);  **Animal model:** 1-methyl-4-phenyl-1,2,3,6-tetrahydropyridine (MPTP)-treated mice | Preclinical | ^206^ |
| **Target lysosomal cathepsins** | **Inhibitor** | E-64 | •Inhibit all cathepsins except cathepsin C | Multiple cell types | Preclinical | ^207^ |
|  |  | FYAD | •Inhibit Cathepsin B and L | IMR-32, SK-N-SH, NB-1691 cells, and GM11027 cells | Preclinical | ^394^ |
|  |  | JPM-OEt | •Inhibit Cathepsin B, L, and S | RIP1-Tag2 (RT2) mouse model of pancreatic islet cell tumorigenesis and MMTV-PyMT-transgenic mouse model. | Preclinical | ^207,395,396^ |
|  |  | CA074 | •Inhibit cathepsin B | Multiple cell types | Preclinical | ^207^ |
|  |  | Nitroxoline | •Inhibit cathepsin B | **Cell type:** breast cancer cell lines: MCF-10A neoT, U-87 MG, HUVEC, and HMEC-1; Mouse breast cancer cell lines: MMTV-PyMT, LPB, and SVEC4-10.  **Animal model:** C57Bl/6 mice bearing LPB fibrosarcoma xenografts and orthotopic mouse breast cancer model. | preclinical | ^397^ |
|  |  | Pepstatin A | •Inhibit cathepsin D | Multiple cell types | Preclinical | ^207^ |
|  |  | JMV4463 | •Inhibit cathepsin D | Human breast (MDA-MB-231 and MCF7), prostate (LNCaP), osteosarcoma (SaOs2) and colorectal (HCT-116) cancer cell lines, and fibroblast cells | Preclinical | ^398^ |
|  |  | Odanacatib | •Inhibit cathepsin K | Multiple cell types | II | ^207,215^ |
|  |  | L-235 | •Inhibit cathepsin K | Intratibial injection model of MDA-MB-231 breast carcinoma in nude rats | Preclinical | ^399^ |
|  |  | Balicatib | •Inhibit cathepsin K | Adult female Macaca fascicularis underwent bilateral ovariectomies | Preclinical | ^400^ |
|  |  | CLIK-148 | •Inhibit cathepsin L | The PC-3 (CRL-1435), U-87 MG (HTB-1), MDA-MB-231 (HTB-26), HOS (CRL-1543), and Raji (CCL-86) cell lines. | Preclinical | ^401^ |
|  |  | KGP94 | •Inhibit cathepsin L | **Cell type:** MDA-MB-231 (human breast cancer cells)  **Animal model:** C3H mouse mammary carcinoma model. | Preclinical | ^402^ |
|  |  | Napsul-Ile-Trp-CHO | •Inhibit cathepsin L | SKN-SH (neuroblastoma) and SaOS2 cells (osteosarcoma). | Preclinical | ^403^ |
|  |  | Z-FL-COCHO | •Inhibit cathepsin S | **Cell type:** human renal carcinoma cell lines: HTB-46, CRL-1611, HTB-44; Human colon carcinoma cell: CCL-247; Mouse kidney cell: TCMK-1.  **Animal model:** mice xenograft model. | Preclinical |  |
|  |  | Fsn0503h | •Inhibit cathepsin S | Multiple cell types | Preclinical | ^404^ |
|  |  | RO5461111 | •Inhibit cathepsin S | MRL-Fas(lpr) mice with SLE and lupus nephritis | Preclinical | ^115^ |
|  |  | RO5459072 | •Inhibit cathepsin S | Enriched Peripheral blood mononuclear cell and cultured RAJI cells | II | ^405^ |
|  | **Enzyme replacement therapy** | AAV | •Adeno-associated virus vector-encoding mouse cathepsin D | *Ctsd-/-* mice | Preclinical | ^108^ |
|  |  | RhCTSD | •Exogenous supplementation of cathepsin D | **Cell type:** mouse embryonic fibroblasts deficient in Cathepsin D.  **Animal model:** *Ctsd-/-* mice. | Preclinical | ^220^ |
| **Target lysosomal membrane permeability and integrity** | **Inducer** | DpdtC | •Evoke the lysosomal ROS generation | HepG2 cells (liver cancer) | Preclinical | ^225^ |
|  |  | Ironomycin  (AM5) | • Promote ROS generation through sequestering iron in lysosomes | Breast cancer stem cells | Preclinical |  |
|  |  | Artemisinin compounds | •Promote ROS generation through enhancing the lysosomal degradation of ferritin | Multiple cell types | I, II | ^228,229^ |
|  |  | ENN B1 | •Destabilize the LAMP-2 complex in the lysosomal membrane | Embryonic fibroblasts | Preclinical | ^230^ |
|  |  | zoledronic acid | •Form stable complexes with the Zn2+ ion resides at the reactive center of the ASM | human follicular lymphoma cells | I, II, III,IV | ^239,406^ |
|  |  | Riccardin D-N | •Inhibit ASM and interfere with sphingomyelin metabolism | **Cell type:** H460 cells (human lung cancer cells).  **Animal model:** Balb/c athymic (nu+/nu+) male mice bearing xenografts of human lung squamous cell carcinoma tissue. | Preclinical | ^234^ |
|  |  | Siramesine | •Inhibit ASM by interfering with its interaction with BMP | **Cell type:** NIH3T3 (fibroblasts), HCT116, Hkh2 (colon cancer), MCF7 (breast cancer), U-2-OS (osteosarcoma), HeLa (cervical cancer), SKOV3 (ovarian cancer), PC3, Du145 (prostate carcinoma).  **Animal model:** female FOX CHASE severe combined immunodeficient (SCID) mice bearing tumor xenografts. | Preclinical | ^235^ |
|  |  | Terfenadine | •Inhibit ASM by interfering with its interaction with BMP | **Cell type:** NIH3T3 (fibroblasts), HCT116, Hkh2 (colon cancer), MCF7 (breast cancer), U-2-OS (osteosarcoma), HeLa (cervical cancer), SKOV3 (ovarian cancer), PC3, Du145 (prostate carcinoma).  **Animal model:** female FOX CHASE severe combined immunodeficient (SCID) mice bearing tumor xenografts. | Preclinical | ^235^ |
|  |  | Amitriptyline | •Detach and inactivate ASM | SY5Y cells (human neuroblastoma cells) | II, III | ^237,407^ |
|  |  | PES (Pifithrin-μ) | •Inhibit Hsp70 though binding its substrate binding domain | BC3 and BCBL1 PEL cell lines, and B lymphocytes | Preclinical | ^240,241^ |
|  |  | Quercetin | •Down-regulate the expression of Hsp70 | Pancreatic cancer cell lines: MiaPaCa-2 and Panc-1; HT29 colon cancer cell line. | I, II, III | ^242^ |
|  |  | Triptolide | •Down-regulate the expression of Hsp70 | Pancreatic cancer cell lines: MiaPaCa-2 and Panc-1; HT29 colon cancer cell line. | I, II | ^242^ |
|  |  | Etoposide | •Upregulate the expression of HspBP1, which bind to Hsp70 | Gastric adenocarcinoma cell lines: MKN1, MKN28, and NUGC-3; Hepatocellular carcinoma cell lines: Hep3B, HLE, HLF; Colon adenocarcinoma cell lines: WiDr, DLD-1; cervical adenocarcinoma cell: HeLa S3; Epidermoid carcinoma cell: A431; Pancreatic adenocarcinoma cell: SUIT-2; Renal cell carcinoma cell: RPMI-SE; Prostatic adenocarcinoma cell: PC3; bladder carcinoma cell: T24; Fibroblasts cell lines: TIG-3, WI-38, and FS-4. | I, II, III, IV | ^243^ |
|  |  | Vincristine | •Destabilize microtubule cytoskeleton | **Cell type:** HeLa (cervical cancer), MCF-7 (breast cancer);  **Animal model:** orthotopic MCF-7 breast cancer xenograft model in mice | I, II, III, IV | ^408^ |
|  |  | Deox B 7,4 | •Target microtubules and vacuolar (V)-ATPase | **Cell type:** leukemia cell lines: TEX, OCI-AML2, K562, THP1, HL60 and U937.  **Animal model:** SCID mice bearing xenograft of OCI-AML-2 cells. | Preclinical | ^137^ |
|  |  | BpV(phen) | •Activate HDAC6 on α-tubulin and impairs stable acetylated microtubules | **Cell type:** cervical cancer (HeLa), liver cancer (HepG2), embryonic fibroblasts (MEFs).  **Animal model:** wild-type mice injected with bpV. | Preclinical | ^257^ |
|  |  | BDE 47 | •Induce ROS production and the loss of mitochondrial membrane potential | HepG2 (liver cancer cell line) | Preclinical | ^256^ |
|  |  | Trehalose | •Regulate autophagy through inducing LMP | NSC34 (mouse motoneuron-like hybrid cell line) | Preclinical | ^258^ |
| **Target lysosomal Calcium signaling** | **Agonist** | ML-SA1 | •Activate TRPML1-3 in lysosomes | HeLa (cervical cancer), HepG2 (liver cancer), 1321 N1 (astrocytoma), HEK293 (human embryonic kidney cells). | Preclinical | ^409,410^ |
|  |  | MK6-83 | •Activate TRPML1, TRPML3 in lysosomes | Glioblastoma cell lines: T98 and U251 cells. | Preclinical | ^268,271^ |
|  |  | ML2-SA1 | •Activate TRPML2 in lysosomes | Murine primary alveolar macrophages | Preclinical | ^272^ |
|  | **Antagonist** | apilimod | •Inhibit PIKfyve in lysosomes | **Cell type:** SU-DHL-6, SU-DHL-10 and WSU-DLCL2 cell lines.  **Animal model:** cancer xenograft model in mice. | Preclinical | ^274^ |
|  |  | YM201636 | •Inhibit PIKfyve in lysosomes | **Cell type:** Liver cancer cell lines: HepG2, Huh‑7, and H22.  **Animal model:** male BALB/c mice injected 2*10^6^ H22 cells subcutaneously. | Preclinical | ^275^ |
|  |  | WX8-family | •Inhibit PIKfyve in lysosomes | U2OS (osteosarcoma), A375 (melanoma), 293T (human embryonic kidney cells), HFF (human foreskin fibroblasts) | Preclinical | ^276^ |
|  |  | Ned-19 | •NAADP antagonist inhibiting TPCs | T24 (bladder carcinoma cell), HUH7 (liver cancer cell), 4T1 (mouse breast cancer cell);  LRRK2-PD fibroblasts. | Preclinical | ^266,277^ |
|  |  | Ned-K | •NAADP antagonist inhibiting TPCs | LRRK2-PD fibroblasts. | Preclinical | ^277^ |
|  |  | Tetrandrine | •Inhibit TPCs in lysosomes | T24 (bladder carcinoma cell), HUH7 (liver cancer cell), 4T1 (mouse breast cancer cell). | Preclinical | ^266^ |
| **Target mTOR signaling** | Rapamycin and its analogs | Sirolimus | •Inhibit mTORC1 | Multiple cell types | I, II, III, IV | ^281^ |
|  |  | Temsirolimus | •Inhibit mTORC1 | Multiple cell types | I, II, III, IV | ^281^ |
|  |  | Everolimus | •Inhibit mTORC1 | Multiple cell types | I, II, III, IV | ^281^ |
|  |  | Ridaforolimus | •Inhibit mTORC1 | Multiple cell types | I, II, III | ^281^ |
|  | Catalytic mTOR inhibitors | Vistusertib (AZD2014) | •Inhibit both mTORC1 and mTORC2 | Multiple cell types | I, II | ^411^ |
|  |  | CC-223 | •Inhibit both mTORC1 and mTORC2 | **Cell type:** hematologic and solid tumor cell lines: PC-3, CAL-51, A549, T47D, NCI-H460, HepG2, AU565, Hep3B, U87MG, HCT116, MDA-MB-231, NCI-H23.  **Animal model:** mice bearing PC-3 tumor xenograft. | I, II | ^412^ |
|  |  | TAK-228  (MLN0128) | •Inhibit both mTORC1 and mTORC2 | Multiple cell types | I, II | ^411^ |
|  | PI3k-AKT inhibitor | Buparlisib (BKM120) | •Pan-class I PI3K inhibitor targeting all four isoforms of p110 | Multiple cell types | I, II, III, | ^281^ |
|  |  | Pictilisib (GDC-0941) | •Pan-class I PI3K inhibitor targeting all four isoforms of p110 | Multiple cell types | I, II | ^281^ |
|  |  | MK-2206 | •Allosteric pan-AKT inhibitor | Multiple cell types | I, II | ^281^ |
|  |  | Capivasertib (AZD5363) | •ATP-competitive pan-AKT inhibitor | Multiple cell types | I, II, III | ^281^ |
|  |  | Ipatasertib (GDC-0068) | •ATP-competitive pan-AKT inhibitor | Multiple cell types | I, II | ^281^ |
|  | Target Ragulator | Knock-out *p18* | • Knock out the essential component of Ragulator resulting the blockade the recruitment of mTORC1 to lysosomes. | **Cell type:** mouse podocytes (MPC5), embryonic fibroblasts.  **Animal model:** mice with p18 deletion specifically in podocytes, podo-Tsc1 KO and podo-Tsc1/p18 DKO mice. | Preclinical | ^285^ |
|  |  | c17orf59 | •Bind to Ragulator to prevent its interaction with the Rag GTPases | HEK 293T cells | Preclinical | ^286^ |
|  | Target Raptor | Nemo-like kinase | •Phosphorylate Raptor to inhibit lysosomal localization of mTORC1 | HEK 293T cells | Preclinical | ^289^ |
|  | Target Rheb | NR1 | •Bind the switch II domain of Rheb | MCF7 (human breast cancer cell line), TRI102 (lymphangioleiomyomatosis derived cell line), and PC3 (human prostate cancer cell line). HEK 293T cells. |  | ^288^ |
| **Emerging targeting strategies** | **Target disturbed lysosomal degradation of immune factors** | PD-LYSO | •Accelerate the lysosomal degradation of PD-L1 | Human colorectal cancer cell lines: HCT116, RKO and LOVO; Pancreatic cancer cell lines: SW1990, PANC1; Melanoma cell line: A375; Breast cancer cell line: MDA-MB-231; Lung cancer cell line A549. T cell. | Preclinical | ^87^ |
|  |  | P22 | •Inhibit PD-L1 and promote the lysosomal degradation of PD-L1 | Co-culture model of Hep3B/OS-8/hPD-L1 and CD3 T cells | Preclinical | ^293^ |
|  |  | SA-49 | •Promote lysosomal biogenesis and induce MITF-dependent lysosomal degradation of PD-L1 | NSCLC cell lines: A549, NCI-H157, NCI-H1975, NCI-H1299, NCI-H460, and Lewis cells; Activated T and NK cells; | Preclinical | ^294^ |
|  |  | CQ + anti-PD1 or anti-CTLA-4 antibodies | • Inhibiting autophagy-lysosomal degradation of MHC-I | **Cell type:** human pancreatic ductal adenocarcinoma cell lines: PaTu-8988T, KP4, MiaPaca2, Panc 2.03, PaTu-8902, Panc1, AsPc1, and HupT3; Human pancreatic duct cell line: HPDE.  **Animal model:** C57BL/6 mice or NCr nude mice used for allograft experiments, OT-I transgenic mice (003831), and Batf3−/− mice (013755) | Preclinical | ^135^ |
|  | **Target TFEB** | Trehalose | •Activates calcium-dependent phosphatase PPP3/calcineurin or inhibits AKT  •Promotes TFEB nuclear translocation | Mouse motoneuron-like hybrid cell line (NSC34); Macrophages isolated from autophagy mouse models. | I, II, III, IV | ^299,413^ |
|  |  | 3,4-DC | •Promote TFEB nuclear translocation though inhibiting mTOR | **Cell type:** HepG2 and U2OS cells;  **Animal model:** wildtype and cardiac-specific Atg7 knockout mice were injected with vehicle control or 3,4-DC; Female GFP-LC3 transgenic mice; Cancer xenograft model in mice. | Preclinical | ^302^ |
|  |  | HPβCD | •Promote TFEB nuclear translocation  •Deplete intracellular cholesterol and inhibit mTORC1 | Fibroblasts derived from patients with LINCL and HeLa cells. | I, II | ^298,303^ |
|  |  | Digoxin | Activate TFEB via Ca^2+^-dependent mechanisms | **Cell type:** HeLa cells and mouse embryonic fibroblasts.  **Animal model:** mouse model for in vivo compound delivery. | I, II, III, IV | ^298^ |
|  | **Target CMA** | P140 | • Reduce the expression level LAMP2A and HSPA8;  • Bind to Hsc70 | Lupus-prone MRL/lpr mice and CBA/J mice | I, II, III | ^310,311^ |
|  | **Target Rab GTPases** | CID1067700 | •Inhibit Rab7 GTPase | **Cell type:** primary astrocytes.  **Animal model:** mice mode of ischemic stroke. | Preclinical | ^315^ |
|  |  | PA | •Targeting RabGGTase | SF21 cells | Preclinical | ^316^ |
|  |  | 3-PEHPC | •Targeting RabGGTase | **Cell type:** peripheral blood lymphocytes isolated from SLE patients and healthy controls; Mouse embryonic fibroblasts.  **Animal model:** MRL/lpr mice. | Preclinical | ^414^ |
|  |  | StRIP3 | •Interfere Rab-GEF interaction | HCT116 | Preclinical | ^317^ |

**Abbreviation**：Ref, reference; CQ, chloroquine; HCQ, hydroxychloroquine; QN, quinacrine; PLGA-aNP, poly(DL-lactide-co-glycolide) acidic nanoparticles; AAV, adeno-associated virus; rhCTSD, recombinant human pro-cathepsin D; rhPPCA, recombinant human protective protein/cathepsin A; ASM, acid sphingomyelinase; ZA, zoledronic acid; rhCTSD, recombinant human pro-Cathepsin D; DpdtC, Di-2-pyridylketone dithiocarbamate; Hsp70, heat shock protein 70; HspBP1, Hsp70 binding protein 1; 3,4-DC, 3,4-dimethoxychalcone; PI(3,5)P2, phosphatidyl-(3,5)-bisphosphate; NSCLC, non-small cell lung cancers; MITF, melanogenesis associated transcription factor; TFEB, transcription factor EB; HPβCD, 2-Hydroxypropyl-β-cyclodextrin; CMA, chaperone-mediated autophagy; PA, psoromic acid; 3-PEHPC, 3-(3-pyridyl)-2-hydroxy-2-phosphonopropanoic acid; RabGGTase, Rab geranylgeranyl transferase. The information provided in the “clinical trial phase” column is the clinical application of the corresponding drug in all diseases.
